# CVIT expert consensus document on primary percutaneous coronary intervention (PCI) for acute coronary syndromes (ACS) in 2024

**DOI:** 10.1007/s12928-024-01036-y

**Published:** 2024-09-20

**Authors:** Yukio Ozaki, Akihiro Tobe, Yoshinobu Onuma, Yoshio Kobayashi, Tetsuya Amano, Takashi Muramatsu, Hideki Ishii, Kyohei Yamaji, Shun Kohsaka, Tevfik F. Ismail, Shiro Uemura, Yutaka Hikichi, Kenichi Tsujita, Junya Ako, Yoshihiro Morino, Yuichiro Maekawa, Toshiro Shinke, Junya Shite, Yasumi Igarashi, Yoshihisa Nakagawa, Nobuo Shiode, Atsunori Okamura, Takayuki Ogawa, Yoshisato Shibata, Takafumi Tsuji, Kentaro Hayashida, Junji Yajima, Teruyasu Sugano, Hiroyuki Okura, Hideki Okayama, Katsuhiro Kawaguchi, Kan Zen, Saeko Takahashi, Toshihiro Tamura, Kazuhiko Nakazato, Junichi Yamaguchi, Osamu Iida, Reina Ozaki, Fuminobu Yoshimachi, Masaharu Ishihara, Toyoaki Murohara, Takafumi Ueno, Hiroyoshi Yokoi, Masato Nakamura, Yuji Ikari, Patrick W. Serruys, Ken Kozuma, The Task Force on Primary Percutaneous Coronary Intervention (PCI) of the Japanese Association of Cardiovascular Intervention, Therapeutics (CVIT)

**Affiliations:** 1 Department of Cardiology, Fujita Health University Okazaki Medical Center, Fujita Health University School of Medicine, 1-98 Dengaku, Kutsukake, Toyoake, Aichi 470-1192 Japan; 2 Department of Cardiology, University of Galway, Galway, Ireland; 3 Department of Cardiovascular Medicine, Chiba University Graduate School of Medicine, Chiba, Japan; 4 Department of Cardiology, Aichi Medical University, Nagakute, Japan; 5 Department of Cardiovascular Medicine, Gunma University Graduate School of Medicine, Maebashi, Japan; 6 Department of Cardiovascular Medicine, Kyoto University Graduate School of Medicine, Kyoto, Japan; 7 Department of Cardiology, Keio University School of Medicine, Tokyo, Japan; 8 King’s College London, London, UK; 9 Guy’s and St Thomas’ Hospital NHS Foundation Trust, London, UK; 10 Cardiovascular Medicine, Kawasaki Medical School, Kurashiki, Japan; 11 Heart Center, Saga Medical Center Koseikan, Saga, Japan; 12 Department of Cardiovascular Medicine, Graduate School of Medical Sciences, Kumamoto University, Kumamoto, Japan; 13 Department of Cardiology, Kitasato University Hospital, Sagamihara, Japan; 14 Department of Cardiology, Iwate Medical University Hospital, Shiwa, Japan; 15 Division of Cardiology, Internal Medicine III, Hamamatsu University School of Medicine, Hamamatsu, Japan; 16 Division of Cardiology, Department of Medicine, Showa University School of Medicine, Tokyo, Japan; 17 Cardiology Division, Osaka Saiseikai Nakatsu Hospital, Osaka, Japan; 18 Division of Cardiology, Sapporo-Kosei General Hospital, Sapporo, Japan; 19 Division of Cardiovascular Medicine, Department of Internal Medicine, Shiga University of Medical Science, Otsu, Japan; 20 Division of Cardiology, Hiroshima City Hiroshima Citizens Hospital, Hiroshima, Japan; 21 Division of Cardiology, Sakurabashi Watanabe Advanced Healthcare Hospital, Osaka, Japan; 22 Division of Cardiology, The Jikei University School of Medicine, Tokyo, Japan; 23 Division of Cardiology, Miyazaki Medical Association Hospital, Miyazaki, Japan; 24 Kusatsu Heart Center, Kusatsu, Japan; 25 Department of Cardiovascular Medicine, The Cardiovascular Institute, Tokyo, Japan; 26 Division of Cardiology, Yokohama City University Medical Center, Yokohama, Japan; 27 Department of Cardiology, Gifu University Graduate School of Medicine, Gifu, Japan; 28 Division of Cardiology, Ehime Prefectural Central Hospital, Matsuyama, Japan; 29 Department of Cardiology, Komaki City Hospital, Komaki, Japan; 30 Department of Cardiovascular Medicine, Kyoto Prefectural University of Medicine, Kyoto, Japan; 31 Division of Cardiology, Tokushukai Shonan Oiso Hospital, Oiso, Japan; 32 Division of Cardiology, Tenri Hospital, Tenri, Japan; 33 Department of Cardiology, Fukushima Medical University Hospital, Fukushima, Japan; 34 Department of Cardiology, Tokyo Women’s Medical University Hospital, Tokyo, Japan; 35 Cardiovascular Division, Osaka Police Hospital, Osaka, Japan; 36 Department of Cardiology, Nagoya University Graduate School of Medicine, Nagoya, Japan; 37 Department of Cardiology, Tokai University Hachioji Hospital, Hachioji, Japan; 38 Department of Cardiovascular and Renal Medicine, Hyogo College of Medicine, Nishinomiya, Japan; 39 Division of Cardiology, Marin Hospital, Fukuoka, Japan; 40 Cardiovascular Center, Fukuoka Sanno Hospital, Fukuoka, Japan; 41 Division of Cardiovascular Medicine, Ohashi Medical Center, Toho University School of Medicine, Tokyo, Japan; 42 Department of Cardiology, Tokai University School of Medicine, Isehara, Japan; 43 Department of Cardiology, Teikyo University Hospital, Tokyo, Japan

**Keywords:** Percutaneous coronary intervention (PCI), Acute coronary syndrome (ACS), ST-segment elevation acute my cardial infarction (STEMI), Non-ST-segment elevation acute myocardial infarction (NSTEMI), Non-ST-segment elevation acute coronary syndrome (NSTE-ACS)

## Abstract

Primary Percutaneous Coronary Intervention (PCI) has significantly contributed to reducing the mortality of patients with ST-segment elevation myocardial infarction (STEMI) even in cardiogenic shock and is now the standard of care in most of Japanese institutions. The Task Force on Primary PCI of the Japanese Association of Cardiovascular Intervention and Therapeutics (CVIT) proposed an expert consensus document for the management of acute myocardial infarction (AMI) focusing on procedural aspects of primary PCI in 2018 and updated in 2022. Recently, the European Society of Cardiology (ESC) published the guidelines for the management of acute coronary syndrome in 2023. Major new updates in the 2023 ESC guideline include: (1) intravascular imaging should be considered to guide PCI (Class IIa); (2) timing of complete revascularization; (3) antiplatelet therapy in patient with high-bleeding risk. Reflecting rapid advances in the field, the Task Force on Primary PCI of the CVIT group has now proposed an updated expert consensus document for the management of ACS focusing on procedural aspects of primary PCI in 2024 version.

## Introduction

In ST-segment elevation myocardial infarction (STEMI), primary PCI has been shown to contribute to the reduction of cardiac events, leads to earlier discharge, and is even effective in patients with cardiogenic shock [1–22]. It is now a standard of care in Japan. CVIT has published an AMI consensus document in 2018 and 2022 to improve the quality of our practice.

Guidelines for management of STEMI and NSTE-ACS were published by the European Society of Cardiology (ESC) in 2017 and 2020, respectively [23, 24]. Recently, the new ESC guidelines for the management of acute coronary syndrome (ACS) were published in 2023 [3]. Major new recommendations are: (1) In high-bleeding risk (HBR) patients, aspirin or P2Y12 receptor inhibitor monotherapy after 1 month of DAPT may be considered (Class IIb, Level B); (2) De-escalation of antiplatelet therapy in the first 30 days after an ACS event is not recommended (Class III, Level B); (3) In patients with spontaneous coronary artery dissection, PCI is recommended only for patients with symptoms and signs of ongoing myocardial ischemia, a large area of myocardium in jeopardy, and reduced antegrade flow (Class I, Level C); (4) Intravascular imaging should be considered to guide PCI. The major revised recommendations are: (1) Routine immediate angiography after resuscitated cardiac arrest is not recommended in hemodynamically stable patients without persistent ST-segment elevation (Class III, Level A); (2) Complete revascularization is recommended either during the index PCI procedure or within 45 days in stable STEMI patients with multivessel disease (Class I, Level A).

However, there are differences between Europe and Japan in available medical devices and drugs as well as healthcare systems, and they may prevent direct application of European guidelines to the Japanese population (Tables 1 and 2). Therefore, the Task Force on PCI of the Japanese CVIT society summarized the consensus for the management of ACS, mainly focusing on procedural aspects.

**Table 1:** Major differences in available medication and mechanical devices

	Europe	Japan
Glycoprotein IIb/IIIa inhibitors	Tirofiban, eptifibatide, and abciximab are available	GP IIb/IIIa inhibitors are not available
P2Y12 inhibitors	The preferred P2Y12 inhibitors are prasugrel [60 mg loading dose and 10 mg maintenance dose once daily per os (p.o.)] or ticagrelor (180 mg p.o. loading dose and 90 mg maintenance dose twice daily)	Both prasugrel and ticagrelor are available, but the dose in prasugrel is different. [20 mg loading dose and 3.75 mg maintenance dose once daily p.o.]
Mechanical LV assist devices	Intra-cardiac axial flow pump (i.e., Impella) and intra-aortic balloon pumping are available	Intra-aortic balloon pumping is in use. Intra-cardiac axial flow pumps are used in selected institutions

**Table 2:** Major CE approved DES and their availability in Japan

DES	Stent platform	Polymer coating	Drug	Strut thickness (μm)	Availability in Japan
Based on durable polymer coatings					
DESyne X2	Cobalt–chrome	PBMA	Novolimus	81	No
EluNIR	Cobalt–chrome	polyurethane	Ridaforolimus	80	Yes
Promus PREMIER	Platinum–chrome	PVDF-HFP	Everolimus	81	No
Resolute Onyx	Cobalt–chrome	BioLinx (PBMA, PHMA, PVP, and PVA)	Zotarolimus	81	Yes
STENTYS	Nitinol	PSU and PVP	Paclitaxel	–	No
Xience Skypoint	Cobalt–chrome	Fluoropolymer (PBMA and PVDF-HFP)	Everolimus	81	Yes
Based on biodegradable polymer coatings					
Biomatrix	Stainless steel	PDLLA	Biolimus A9	120	No
BioMime	Cobalt–chrome	PLLA and PLGA	Sirolimus	65	No
Combo	Stainless steel	PDLLA and PLGA + Additional coating with anti- CD34	Sirolimus	102	Yes
DESyne BD	Cobalt–chrome	PLLA	Novolimus	81	No
DynamX	Cobalt–chrome	PLLA and PLGA	Novolimus	71	No
HT Supreme	Cobalt–chrome	PLGA	Sirolimus	80	No
MiStent	Cobalt–chrome	PLGA	Crystalline sirolimus	64	No
Orsiro mission	Cobalt–chrome	PLLA	Sirolimus	60	Yes
Supraflex Cruz	Cobalt–chrome	PLLA, PLGA, and PVP	Sirolimus	60	No
Supralimus Core	Cobalt–chrome	PLLA, PLGA, PCL, and PVP	Sirolimus	60	No
SYNERGY	Platinum–chrome	PLGA	Everolimus	74	Yes
Ultimaster Nagomi	Cobalt–chrome	PDLLA and PCL	Sirolimus	80	Yes
Yukon Chorome PC	Cobalt–chrome	PDLLA	Sirolimus	68	No
Polymer-free					
Amazonia Pax	Cobalt–chrome	–	Paclitaxel	73	No
BioFreedom Ultra	Stainless steel	–	Biolimus A9	84	Yes
Cre8 EVO	Cobalt–chrome	–	Sirolimus	70	No

*CE* Conformité Européenne; *DES* drug eluting stent; *PBMA* poly-*n*-butyl methacrylate; *PCL* poly-caprolactone; *PDLLA* poly-d,l-lactic acid; *PHMA* poly-hexyl methacrylate; *PLCL* poly-l-lactide-*co*-caprolactone; *PLGA* poly-d,l-lactide-*co*-glycolide; *PLLA* poly-l-lactic acid; *PVP* polyvinylpyrrolidone; *PVA* polyvinyl acetate; *PVDF-HFP* poly-vinylidene fluoride-*co*-hexafluoropropylene; *PSU* polysulphone

### Specific differences between Japan and Europe

Glycoprotein (GP) IIb/IIIa inhibitors are not available in Japan, and therefore, thrombus aspiration remains a choice of strategy of treatment for ACS with thrombotic lesions.

Currently preferred oral P2Y12 inhibitors in acute coronary syndrome in Europe are prasugrel and ticagrelor, and both are also available in Japan. However, dose differences in P2Y12 inhibitors between Japan and Europe may cause different antithrombotic benefit/bleeding risk profile. In general, Asian patients are at higher risk of bleeding but at lower risk of thrombotic events when compared to Western patients [25]. Due to the genetic variance in the CYP450, clopidogrel metabolism may vary among East Asian populations, potentially resulting in less potent effect of clopidogrel in Asian than in Western populations [26]. Intravenous cangrelor and subcutaneous selatogrel are not approved in Japan, while their use may be considered in patients not pre-treated with oral P2Y12 inhibitors at the time of PCI or in those who are considered unable to absorb oral agents.

Impella, an intra-cardiac axial flow left-ventricular assist device, and extracorporeal membrane oxygenation (ECMO) are increasingly popular for managing patients with cardiogenic shock in Europe, although they have not been sufficiently evaluated in clinical trials [27], while the use of intra-aortic balloon pump (IABP) has not met prior expectations of benefit [23, 28]. In Japan, Impella became available in 2017 and its use is increasing year by year; however, we still largely rely on IABP as a mechanical support.

Regarding intravascular imaging devices, intravascular ultrasound (IVUS) and optical coherence tomography (OCT) during PCI are routinely reimbursed in Japan. In contrast to the situation in Europe, their use is not restricted in Japan.

**Fig. 1:**
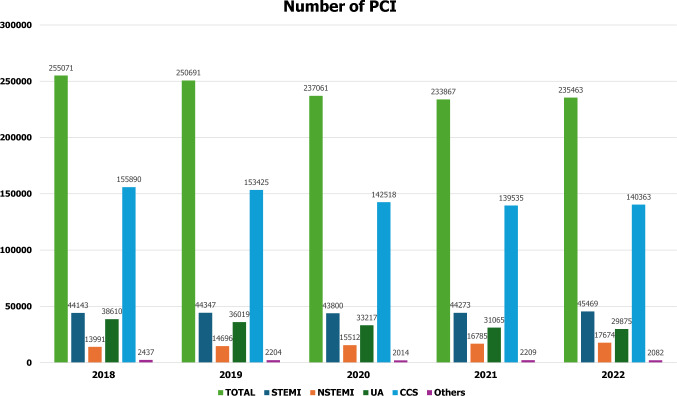
The annual number of PCI from 2018 to 2022. The data are based on the J-PCI registry. *STEMI* ST-segment elevated myocardial infarction; *NSTEMI* non-ST-segment elevation myocardial infarction; *UA* unstable angina; *CCS* chronic coronary syndrome. CCS includes stable angina, asymptomatic ischemia, chronic total occlusion, and staged PCI

**Fig. 2:**
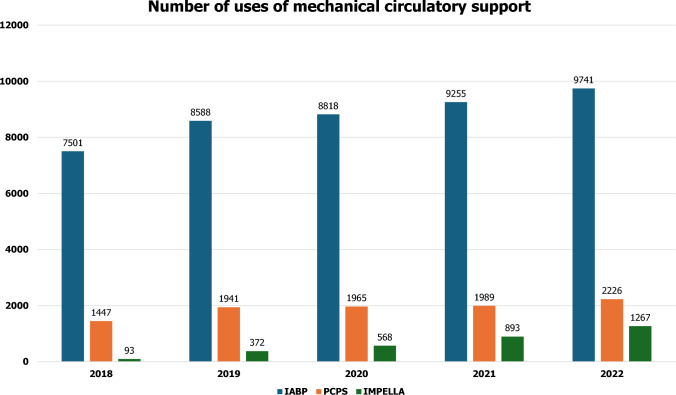
The annual number of uses of mechanical circulatory support from 2018 to 2022. The data are based on the J-PCI registry. *IABP* intra-aortic balloon pumping; *PCPS* percutaneous cardiopulmonary support

In terms of data derived from the Japanese population, there are several registries and databases including patients with AMI in Japan, such as J-MINUET [29], PACIFIC [30], Tokyo CCU network registry [31], JAMIR [32, 33], and JROAD [34–36]. CVIT has been working on the J-PCI registry [37–41], the largest database of patients who underwent PCI in Japan. Figures 1 and 2 show the annual number of PCI and use of mechanical circulatory support, respectively, registered in J-PCI registry from 2018 to 2022. The demographics, lesion, and procedural characteristics in patients with ACS, registered in J-PCI registry in 2022—235,463 patients treated in 1173 institutions are listed in Tables 3 and 4. There were 93,018 ACS patients in total and the rate of procedural success, which was defined as visually assessed residual stenosis in the target-vessel of less than 25%, and no delay in contrast reaching the distal vessels with TIMI 3 flow was 97%, and the in-hospital mortality was 4.3%. The door-to-balloon time in STEMI patients was 87 ± 54 min.

**Table 3:** Demographics of patients with ACS from J-PCI registry in 2022

	Overall ACS	STEMI	NSTEMI	UA
	(*N* = 93,018)	(*n* = 45,469)	(*n* = 17,674)	(*n* = 29,875)
Age (years), mean (± SD)	71.0 ± 12.4	70.2 ± 12.9	71.8 ± 12.4	71.8 ± 11.6
Male	70,467/93,018 (75.8%)	34,580/45,469 (76.1%)	13,286/17,674 (75.2%)	22,601/29,875 (75.7%)
Cardiogenic shock	8161/92,349 (8.8%)	6252/45,350 (13.8%)	1345/17,628 (7.63%)	564/29,371 (1.92%)
*Risk factors*				
Smoker (current and within 1-yr)	30,661/88,464 (34.7%)	16,529/42,726 (38.7%)	5718/16,928 (33.8%)	8414/28,810 (29.2%)
Diabetes mellitus	37,534/88,464 (42.4%)	16,945/42,726 (39.7%)	7511/16,928 (44.4%)	13,078/28,810 (45.4%)
Hypertension	67,772/88,464 (76.6%)	31,341/42,726 (73.4%)	13,339/16,928 (78.8%)	23,092/28,810 (80.2%)
Hypercholesterolemia	58,368/88,464 (66.0%)	26,552/42,726 (62.1%)	11,420/16,928 (67.5%)	20,396/28,810 (70.8%)
*History of*				
Previous MI	13,817/91,626 (15.1%)	4,716/44,972 (10.5%)	3365/17,498 (19.2%)	5736/29,156 (19.7%)
Peripheral vascular disease	4304/88,464 (4.9%)	1448/42,726 (3.4%)	956/16,928 (5.7%)	1900/28,810 (6.6%)
Previous PCI	22,445/92,157 (24.4%)	6144/45,205 (13.6%)	4523/17,619 (25.7%)	11,778/29,333 (40.2%)
Previous CABG	2146/92,141 (2.3%)	443/45,225 (1.0%)	551/17,612 (3.1%)	1152/29,304 (3.9%)
Heart failure	10,215/91,360 (11.1%)	3129/44,766 (7.0%)	2577/17,462 (14.8%)	4509/29,132 (15.5%)
Renal insufficiency	21,961/88,464 (24.8%)	9684/42,726 (22.7%)	4764/16,928 (28.1%)	7513/28,810 (26.1%)
Hemodialysis	4524/88,464 (5.1%)	1130/42,726 (2.6%)	1091/16,928 (6.4%)	2303/28,810 (8.0%)
Chronic lung disease (COPD)	2749/88,464 (3.1%)	1246/42,726 (2.9%)	618/16,928 (3.7%)	885/28,810 (3.1%)
COVID-19	587/88,464 (0.6%)	354/42,726 (0.8%)	119/16,928 (0.7%)	114/28,810 (0.4%)
*Antiplatelet prescribed before or at procedure*				
Any antiplatelet prescribed	81,691/92,974 (87.9%)	38,214/45,433 (84.1%)	15,677/17663 (88.8%)	27,800/29,868 (93.1%)
Aspirin	78,065/81,866 (95.4%)	36,771/38,314 (96.0%)	14,945/15,713 (95.1%)	26,349/27,839 (94.6%)
Clopidogrel	13,828/81,866 (16.9%)	3573/38,314 (9.33%)	2689/15,713 (17.1%)	7566/27,839 (27.2%)
Prasugrel	60,928/81,866 (74.4%)	31,595/38,314 (82.5%)	11,575/15,713 (73.7%)	17,758/27,839 (63.8%)
Ticagrelor	189/81,866 (0.2%)	88/38,314 (0.2%)	37/15,713 (0.2%)	64/27,839 (0.2%)
*Oral anticoagulant prescribed before or at procedure*				
Any anticoagulation prescribed	5106/93,018 (5.5%)	1804/45,469 (4.0%)	1147/17,674 (6.5%)	2155/29,875 (7.2%)
Warfarin	1253/5267 (23.8%)	478/1889 (25.2%)	304/1176 (25.9%)	471/2,202 (21.4%)
DOAC	3748/5267 (71.2%)	1308/1889 (69.2%)	820/1176 (69.7%)	1620/2202 (73.6%)
Door to balloon time, min	NA	87.1 ± 53.6 (*n* = 38,142)	NA	NA
In-hospital mortality	4039/93,018 (4.3%)	2924/45,469 (6.4%)	734/17,674 (4.2%)	381/29,875 (1.3%)

Data are counts (percentage) unless otherwise specified

*CABG* coronary artery bypass grafting; *DOAC* direct oral anticoagulants; *MI* myocardial infarction; *NSTEMI* non ST-elevation myocardial infarction; *PCI* percutaneous coronary intervention; *STEMI* ST-elevation myocardial infarction

**Table 4:** Lesion and procedural characteristics in patients with ACS from J-PCI registry in 2022

	Overall ACS	STEMI	NSTEMI	UA
	(*N* = 93,018)	(*n* = 45,469)	(*n* = 17,674)	(*n* = 29,875)
*Lesion characteristics*				
Single vessel disease	55,288/93,018 (59.4%)	28,006/45,469 (61.6%)	9273/17,674 (52.5%)	18,009/29,875 (60.3%)
Double vessel disease	22,094/93,018 (23.8%)	10,435/45,469 (22.9%)	4612/17,674 (26.1%)	7047/29,875 (23.6%)
Triple vessel disease	11,755/93,018 (12.6%)	5463/45,469 (12.0%)	2810/17,674 (15.9%)	3482/29,875 (11.7%)
LMT	717/93,018 (0.8%)	340/45,469 (0.7%)	164/17,674 (0.9%)	213/29,875 (0.7%)
Procedure details				
*Approach*				
Transfemoral	17,009/93,018 (18.3%)	9441/45,469 (20.8%)	2998/17,674 (17.0%)	4570/29,875 (15.3%)
Transradial	72,820/93,018 (78.3%)	34,854/45,469 (76.7%)	14,085/17,674 (79.7%)	23,881/29,875 (79.9%)
Others	3189/93,018 (3.4%)	1174/45,469 (2.6%)	591/17,674 (3.3%)	1424/29,875 (4.8%)
*Device*				
DCB	15,326/93,018 (16.5%)	4834/45,469 (10.6%)	3497/17,674 (19.8%)	6995/29,875 (23.4%)
DES	76,734/93,018 (82.5%)	38,679/45,469 (85.1%)	14,135/17,674 (80.0%)	23,920/29,875 (80.1%)
BMS	179/93,018 (0.2%)	82/45,469 (0.2%)	24/17,674 (0.1%)	73/29,875 (0.2%)
Balloon	28,983/93,018 (31.2%)	14,383/45,469 (31.6%)	5561/17,674 (31.5%)	9039/29,875 (30.3%)
BRS	62/93,018 (0.07%)	37/45,469 (0.08%)	10/17,674 (0.06%)	15/29,875 (0.05%)
Thrombus aspiration	23,211/93,018 (25.0%)	19,539/45,469 (43.0%)	2556/17,674 (14.5%)	1116/29,875 (3.7%)
Distal protection	3623/93,018 (3.9%)	2357/45,469 (5.2%)	516/17,674 (2.9%)	750/29,875 (2.5%)
*Mechanical circulatory support*				
IABP	8133/93,018 (8.7%)	5697/45,469 (12.5%)	1493/17,674 (8.5%)	943/29,875 (3.2%)
PCPS	1958/93,018 (2.1%)	1459/45,469 (3.2%)	362/17,674 (2.1%)	137/29,875 (0.5%)
Impella	1063/93,018 (1.1%)	781/45,469 (1.7%)	182/17,674 (1.0%)	100/29,875 (0.3%)
Procedural success^a^	90,403/93,018 (97.2%)	44,211/45,469 (97.2%)	17,051/17,674 (96.5%)	29,141/29,875 (97.5%)

Data are counts (percentage)

*BMS* bare-metal stent; *DES* drug-eluting stent; *IABP* intra-aortic balloon pumping; *MI* myocardial infarction; *NSTEMI* non ST-elevation myocardial infarction; *PCPS* percutaneous cardio pulmonary support; *STEMI* ST-elevation myocardial infarction; *TIMI* thrombolysis in myocardial infarction

^a^Procedural success is defined as visually assessed residual stenosis in the target vessel of less than 25%, and no delay in contrast reaching the distal vessels with TIMI 3 flow

### Work style reform of physicians in Japan

In Japan, work style reform of physicians has started since April 2024 aiming to improve the physicians’ working environment. Now, physicians’ working hours are regulated by law to reduce overworking. Since physicians are workers, they are subject to the Labor Standards Act, which stipulates the principle of working hours of 8 h per day and 40 h per week. However, if it is unavoidable to exceed this principle, it is necessary to conclude an agreement between workers and management in accordance with Article 36 of the Labor Standards Act. The maximum hours of overtime vary depending on the applicable level. In principle, the maximum is 960 h per year, but under certain circumstances, it can be up to 1860 h per year. However, there is also a limit of 100 h per month, and if this is exceeded, an interview with an occupational health physician is required. While there is an expectation that these changes in the system will enable the provision of sustainable healthcare, there are several concerns, especially in the practice of cardiology, which handles a high number of emergency cases. A particular concern is that the regional disparities in healthcare might widen. The door-to-balloon time in STEMI patients was reportedly longer in rural area than in urban area even before the work style reform had started [32]. A study using JROAD-DPC (the Japanese Registry of All cardiac and vascular Disease-Diagnostic Procedure Combination) reported that higher hospital density and larger numbers of cardiologists in the hospital are related with a lower mortality in AMI patients [34]. We need to carefully observe the impact of work style reform on the practice of cardiology, especially on regional healthcare.

### COVID-19 pandemic

The COVID-19 pandemic significantly affected acute cardiac care all over the world [42, 43]. In Japan, the first “state of emergency” declaration was issued from April 7 to May 25, 2020. A study from the J-PCI registry reported a reduction in the number of STEMI patients who underwent PCI as well as total PCI patients in 2020 compared to 2019 (Fig. 1), especially in April and May [37]. More patients had high-risk features in 2020, which might be due to patients’ hesitation to visit healthcare institutes or restricted access to them. Higher crude and adjusted in-hospital mortality were observed in the total PCI population in 2020 than in 2019. However, in STEMI patients, there was no significant difference in the adjusted in-hospital mortality as well as the door-to-balloon time between the two durations. The unique healthcare system in Japan may have contributed to the treatment of STEMI patients, even under the COVID-19 pandemic.

## Primary PCI in STEMI, immediate/early invasive versus conservative strategy in NSTEMI

### Primary PCI in STEMI

Primary PCI for STEMI has been shown to contribute to high revascularization success rates, less cardiac events, and earlier discharge, and is even effective in patients with cardiogenic shock; however, its clinical benefit is time sensitive. The 2023 ESC guidelines recommend reperfusion therapy (primary PCI or fibrinolysis) in patients with STEMI within 12 h of onset, and primary PCI is preferred over fibrinolysis if PCI can be initiated within 120 min from diagnosis (class I). Compared to myocardial infarction within 12 h of onset, there is less evidence on benefit of primary PCI regarding myocardial infarction occurring after 12 h from onset. Recently, Bouisset et al. analyzed three nationwide registries from the FAST-MI (French Registry of Acute ST-elevation and non-ST-elevation Myocardial Infarction). In 1077 STEMI patients admitted 12–48 h from symptom onset, revascularization within 48 h after hospital admission was associated with reduced rates of all-cause mortality at 30 days and long-term follow-up [44]. A primary PCI strategy in STEMI patients presenting 12–48 h after symptom onset is recommended as class IIa in 2023 ESC guidelines. The Occluded Artery Trial (OAT) investigated the effect of PCI in addition to optimal medical therapy (OMT) in 2166 stable patients with occluded infarct-related artery 3–28 days after onset of MI. PCI plus OMT did not demonstrate clinical benefits compared to OMT alone during 4-year follow-up [45]. Routine PCI for occluded infarct-related artery in STEMI patients presenting > 48 h after symptom onset without persistent symptom is not recommended in the 2023 ESC guideline (class III).

### Invasive strategy in NSTE-ACS

An immediate invasive strategy, which refers to as soon as possible angiography and PCI if indicated, is recommended as a class I indication in the 2023 ESC guideline for NSTE-ACS patients who meet one or more of the following very high-risk criteria: hemodynamic instability or cardiogenic shock; recurrent or refractory chest pain despite medical treatment; life-threatening arrhythmias; mechanical complications of MI; HF clearly related to ACS; and recurrent dynamic ST-segment or T-wave changes, particularly with intermittent ST-segment elevation.

A routine invasive strategy is recommended for NSTE-ACS patients. Meta-analysis based on individual-patient data from three studies (FRISC-II, ICTUS, and RITA-3) that compared a routine invasive against a selective invasive strategy in NSTE-ACS patients revealed lower rates of cardiovascular death and myocardial infarction at 5-year follow-up (HR 0.81; 95% CI 0.71–0.93; *P* = 0.002), with the most pronounced difference in high-risk patients [46]. Age, diabetes, previous myocardial infarction, ST-segment depression, hypertension, body mass index (< 25 or > 35 kg/m^2^), and treatment strategy were found to be independent predictors of death and myocardial infarction during follow-up. The results support a routine invasive strategy but highlight the importance of risk stratification in the decision-making process as is recommended in the present guidelines.

An early invasive strategy, which refers to routine invasive angiography and PCI if needed within 24 h of presentation, is recommended as a class IIa indication in 2023 ESC guidelines for NSTE-ACS patients who meet any of the following high-risk criteria: confirmed NSTEMI based on the hs-cTn-based ESC algorithm; dynamic ST-segment or T-wave changes; transient ST-segment elevation; or a GRACE score > 140. In the VERDICT trial, 2147 patients with NSTE-ACS were randomized to invasive coronary angiography within 12 h or standard invasive care within 48–72 h [47]. Overall, early invasive coronary angiography did not improve the primary endpoint at 5 years (all-cause death, nonfatal AMI, hospital admission for refractory myocardial ischemia, or hospital admission for heart failure; HR 0.92, 95% CI 0.78–1.08). However, in patients with a GRACE risk score > 140, early invasive coronary angiography significantly reduced the primary endpoint (HR 0.81, 95% CI 0.66–0.99). In addition, early invasive coronary angiography had some benefits in patients with troponin elevation (i.e., NSTEMI) and ST-T change (HR 0.85, 95% CI 0.71–1.01; and HR 0.80, 95% CI 0.63–1.01, respectively).

The GRACE risk score was applied to the patients with acute coronary syndromes (ACS) in the Tokyo CCU (cardiovascular care unit) Network Database. A total of 9460 patients with ACS hospitalized at 67 Tokyo CCUs were retrospectively reviewed and there was a strong correlation between the GRACE risk score and in-hospital mortality for patients with STEMI or NSTEMI (*r* = 0.99, *P* < 0.001); however, the correlation was not significant for patients with unstable angina (*r* = 0.35, *P* = 0.126). Furthermore, a J-MINUET substudy examining the impact of chronic kidney disease (CKD) on long-term outcomes in 3,281 Japanese patients with AMI demonstrated that 3-year mortality and MACE significantly deteriorated from 5.09% and 15.8% in no-CKD through 16.3% and 38.2% in moderate-CKD to 36.7% and 57.9% in severe-CKD, respectively (*P* < 0.0001) [48]. CKD remains a useful predictor of in-hospital and 3-year mortality as well as MACE after AMI in the modern PCI and optimal medical therapy era [48]. We recommend the use of the GRACE score to identify high-risk patients with AMI [49].

In cases of cardiac arrest without STEMI, the Coronary Angiography After Cardiac Arrest (COACT) trial compared immediate angiography with an intent to revascularize with delayed angiography in patients who successfully resuscitated after out-of-hospital cardiac arrest and had no signs of STEMI [50]. Immediate angiography did not reduce mortality, compared to the delayed angiography group [51].

### Recommendations

- Primary PCI of the infarct-related artery (IRA) is recommended in STEMI.
- An immediate invasive strategy (as soon as possible) is recommended in NSTE-ACS patients at very high risk (hemodynamic instability or cardiogenic shock, recurrent or refractory chest pain despite medical treatment, life-threatening arrhythmias, mechanical complications of MI, HF clearly related to ACS, and recurrent dynamic ST-segment or T-wave changes, particularly with intermittent ST-segment elevation).
- An early invasive strategy (< 24 h) is recommended in NSTE-ACS patients with at high risk (confirmed NSTEMI based on the hs-cTn-based ESC algorithm, dynamic ST-segment or T-wave changes, transient ST-segment elevation, or a GRACE score > 140).

## Practical recommendation for primary PCI

### Loading dose DAPT

Prasugrel and ticagrelor reduce ischemic events and mortality in ACS patients compared to clopidogrel and are recommended by the current guidelines [23, 24, 52].

In TRITON-TIMI 38, 13,608 patients with ACS with scheduled PCI were randomized to either prasugrel or clopidogrel. Prasugrel therapy was associated with significantly reduced rates of ischemic events, including stent thrombosis, but with an increased risk of major bleeding, including fatal bleeding. Overall mortality did not differ significantly between treatment groups [52]. In the Japanese population, the PRASFIT-ACS study was conducted to confirm the efficacy and safety of prasugrel at loading/maintenance doses of 20/3.75 mg [53]. Japanese patients (*n* = 1363) with ACS undergoing PCI were randomized to either prasugrel (20 mg for loading/3.75 mg for maintenance) or clopidogrel (300 mg for loading/75 mg for maintenance). The incidence of MACE at 24 weeks was 9.4% in the prasugrel group and 11.8% in the clopidogrel group (risk reduction 23%, hazard ratio 0.77, 95% confidence interval 0.56–1.07). The incidence of non-coronary artery bypass graft-related major bleeding was similar in both groups (1.9% vs 2.2%). The results were similar to TRITON-TIMI 38 with a low risk of clinically serious bleeding in Japanese ACS patients.

Regarding ticagrelor, clinical outcomes in a large real-world post-ACS population were studied in a Swedish prospective cohort study of 45,073 ACS patients who were discharged on ticagrelor (*N* = 11,954) or clopidogrel (*N* = 33,119) [54]. The risk of the primary outcome (composite of all-cause death, re-admission with Ml, or stroke) with ticagrelor vs clopidogrel was 11.7% vs 22.3% (adjusted HR (HR) 0.85 [95% Cl 0.78–0.93]), risk of death 5.8% vs 12.9% (adjusted HR 0.83 [0.75–0.921], and risk of MI 6.1% vs 10.8% (adjusted HR 0.89 [0.78–1.011] at 24 months. Re-admission rates for bleeding with ticagrelor versus clopidogrel were similar. Both ticagrelor and clopidogrel post-ACS were associated with a lower risk of death, Ml, or stroke, as well as death alone. The risk of bleeding was higher with ticagrelor [54]. These real-world outcomes are consistent with the results of the landmark Platelet Inhibition and Patient Outcomes (PLATO) trial [55].

The ISAR-REACT 5 trial compared prasugrel plus aspirin vs ticagrelor plus aspirin in 4018 ACS patients [56]. The trial demonstrated that treatment with prasugrel, compared to ticagrelor, significantly reduced the composite rate of death, MI, or stroke (6.9% vs 9.3%, *P* = 0.006) without any increase in bleeding complications (4.8% vs 5.4%, *P* = 0.46).

Both prasugrel and ticagrelor are available for clinical use in Japan. The recommended dose of ticagrelor is the same as in Europe and United Sates of America, while the dose of prasugrel was reduced according to the PRASFIT-ACS study in Japan [53] (EU: 60 mg loading dose and 10 mg maintenance dose once daily; Japan: 20 mg loading dose and 3.75 mg maintenance dose once daily) (Table 1).

### Evidence from Japan

#### ASET-Japan

The ASET (Acetyl Salicylic Elimination Trial)-JAPAN pilot study was designed to investigate the feasibility of prasugrel monotherapy with Japanese adjusted dose (3.75 mg/day) after SYNERGY stent implantation in Japanese patients with chronic coronary syndrome (CCS) or NSTE-ACS with anatomical SYNTAX Score < 23 [57]. Patients were loaded with standard DAPT (aspirin 81–330 mg and prasugrel 20 mg) if they were not on long-term DAPT (≧5 days prior to the index PCI). After PCI, patients received prasugrel monotherapy (3.75 mg/day) until 3-month follow-up in CCS cohort and until 12-month follow-up in NSTE-ACS cohort. The primary outcome at 3 months in 206 CCS patients showed the safety and feasibility of prasugrel monotherapy; there were no primary bleeding (BARC type 3 or 5) or ischemic (a composite of cardiac death, spontaneous target-vessel MI, or definite stent thrombosis) events [58]. The 1-month result of NSTE-ACS cohort (*n* = 101) was presented at EuroPCR 2024, and there were no primary ischemic events and two primary bleeding events. The 1-year results of NSTE-ACS cohort will be presented soon.

#### STOPDAPT-3

The STOPDAPT-3 trial randomized 6002 patients with ACS or HBR either to prasugrel monotherapy (3.75 mg/day) or to DAPT (aspirin and prasugrel) after Xience stent implantation in Japan. In both groups, 20 mg of prasugrel was loaded. In DAPT group, aspirin (162–200 mg) was also loaded if the patients were aspirin naive. The prasugrel monotherapy group received 3.75 mg/day of prasugrel until 1-month follow-up. In this trial, 75% of the patients had ACS, and 43% were patients with STEMI. The prasugrel monotherapy was non-inferior to DAPT with regard to the coprimary cardiovascular endpoint (4.12% vs 3.69%; hazard ratio 1.12 [0.87–1.45]; *P*_non-inferiority_ = 0.01), however, failed to show the superiority for coprimary bleeding endpoint (4.47% vs 4.71%; hazard ratio 0.95 [0.75–1.20]; *P*_superiority_ = 0.66) at 1 month. The incidence of unplanned coronary revascularization and subacute definite or probable stent thrombosis were higher in the prasugrel monotherapy group [59]. The prasugrel monotherapy with Japanese adjusted dose (3.75 mg/day) seems to have no benefit over 1-month DAPT in terms of reduction of bleeding after DES implantation in patients with ACS or HBR.

### Recommendations

- Recommended dose of aspirin: 162–325 mg loading dose and 81–162 mg maintenance dose once daily per os.
- Recommended dose of prasugrel: 20 mg loading dose and 3.75 mg maintenance dose once daily per os.
- Recommended dose of ticagrelor: 180 mg p.o. loading dose and 90 mg maintenance dose twice daily.

### Anticoagulation during PCI

Routine use of unfractionated heparin (UFH) is recommended as a class I indication and alternative use of enoxaparin or bivalirudin during primary PCI or early invasive angiography is a class IIa recommendation in the 2023 ESC ACS guidelines.

There has been no placebo-controlled trial evaluating UFH in primary PCI, but there is a large body of experience with this agent. Dosage should follow standard recommendations for PCI (i.e., initial bolus 70–100 U/kg). There are no robust data recommending the use of activated clotting time to tailor dose or monitor UFH, and if activated clotting time is used, it should not delay recanalization of the artery.

An intravenous bolus of enoxaparin 0.5 mg/kg was compared with UFH in the ATOLL randomized trial including 910 STEMI patients [60]. The primary composite endpoint of 30-day death, MI, procedural failure, or major bleeding was not significantly reduced by enoxaparin (17% relative risk reduction, *P* = 0.063), but there was a reduction in the composite main secondary endpoint of death, recurrent MI or ACS, or urgent revascularization. Importantly, there was no evidence of increased bleeding following the use of enoxaparin over UFH. In a meta-analysis of 23 PCI trials (30,966 patients, 33% primary PCI), enoxaparin was associated with a significant reduction in death compared to UFH. This effect was particularly significant in the primary PCI context and was associated with a reduction in major bleeding [61]. In Japan, enoxaparin is approved only for subcutaneous administration and is practically difficult to use during PCI.

A meta-analysis comparing bivalirudin with UFH with or without planned use of GP IIb/IIIa inhibitors in patients with STEMI trials showed no mortality advantage with bivalirudin and a reduction in the risk of major bleeding, but at the cost of an increased risk of acute stent thrombosis [62]. In the recent MATRIX trial including 7213 ACS patients (56% with STEMI), bivalirudin did not reduce the incidence of the primary endpoint (composite of death, MI, or stroke) compared to UFH. Bivalirudin was associated with lower total and cardiovascular mortality, lower bleeding, and more definite stent thrombosis [63]. A post-hoc analysis suggested that prolonging bivalirudin with a full-PCI dose after PCI was associated with the lowest risk of ischemic and bleeding events, which is in accordance with the current labeling of the drug [63]. Bivalirudin could be considered in STEMI, especially in patients at high-bleeding risk [64–66]. Bivalirudin is recommended for patients with heparin-induced thrombocytopenia.

After the publication of the 2017 ESC guidelines, the VALIDATE-SWEDEHEART (Bivalirudin versus Heparin in ST-Segment and Non-ST-Segment Elevation Myocardial Infarction in Patients on Modern Antiplatelet Therapy in the Swedish Web System for Enhancement and Development of Evidence-based Care in Heart Disease Evaluated according to Recommended Therapies Registry Trial) multicenter, randomized, registry-based trial was published [67]. Patients with either ST-segment elevation MI (*N* = 3,005) or non-ST-segment elevation MI (*N* = 3001) undergoing PCI and receiving a potent P2Y12 inhibitor (ticagrelor, prasugrel, or cangrelor) without the planned use of glycoprotein IIb/IIIa inhibitors were randomly assigned to receive bivalirudin or heparin during PCI, performed predominantly with the use of radial artery access. The primary composite endpoint (death from any cause, MI, or major bleeding during 180 days of follow-up) occurred in 12.3% of the patients in the bivalirudin group and in 12.8% in the heparin group (HR 0.96; 95% CI 0.83 to 1.10; *P* = 0.54). The results were consistent between patients with ST-segment elevation MI and those with non-ST-segment elevation MI and across other major subgroups. There was no difference between groups in MI, major bleeding, definite stent thrombosis or mortality. This study shows overall clinical non-inferiority for the use of bivalirudin or heparin during PCI for ACS, along with increased cost with use of bivalirudin. Thus, the use of bivalirudin during PCI was downgraded to a class IIb recommendation. Consistent with these findings, the current uptake of bivalirudin in Europe is very low. Bivalirudin remains unavailable in Japan with no evaluation by clinical trials.

Glycoprotein (GP) IIb/IIIa inhibitors are the strongest antiplatelet agents currently available in Europe and in the US but remain unavailable in Japan. There are three different compounds, namely abciximab, tirofiban, and eptifibatide. However, procedural use of abciximab plus unfractionated heparin (UFH) showed no benefit compared to bivalirudin [64]. In Japan, JEPPORT a randomized placebo-controlled trial (*n* = 973), abciximab did not show efficacy in reducing the primary endpoint (30-day post-PCI coronary events: death, MI, or urgent revascularization) [68]. However, using GP IIb/IIIa inhibitors as bailout therapy in the event of angiographic evidence of a large thrombus, slow- or no-reflow, and other thrombotic complications is reasonable, as recommended in 2017 ESC guidelines [23], although this strategy has not been tested in a randomized trial. Overall, there is no evidence to recommend the routine use of GP IIb/IIIa inhibitors for primary PCI.

### Recommendations

- Anticoagulation is recommended for all patients in addition to antiplatelet therapy during primary PCI.
- Routine use of UFH is recommended.

### Approach (femoral vs radial)

Over recent years, several studies have provided robust evidence in favor of the radial approach as the default access site in ACS patients undergoing primary PCI by experienced radial operators [69, 70]. In the Minimizing Adverse Hemorrhagic Events by TRansradial Access Site and Systemic Implementation of angioX (MATRIX) program, patients were randomized to radial or femoral access, stratified by STEMI (2001 radial, 2009 femoral) and NSTE-ACS (2196 radial, 2198 femoral). MACE occurred in 121 (6.1%) STEMI patients with radial access vs 126 (6.3%) patients with femoral access [rate ratio (RR) = 0.96, 95% CI = 0.75–1.24; *P* = 0.76] and in 248 (11.3%) NSTE-ACS patients with radial access vs 303 (13.9%) with femoral access (RR = 0.80, 95% CI 0.67–0.96; *P* = 0.016) (*P*_int_ = 0.25). MACE occurred in 142 (7.2%) STEMI patients with radial access and in 165 (8.3%) patients with femoral access (RR = 0.86, 95% CI 0.68–1.08; *P* = 0.18) and in 268 (12.2%) NSTE-ACS patients with radial access compared with 321 (14.7%) with femoral access (RR = 0.82, 95% CI 0.69–0.97; *P* = 0.023) (*P*_int_ = 0.76). All-cause mortality and access site-actionable bleeding favored radial access irrespective of ACS type (*P*_interaction_ = 0.11 and *P*_interaction_ = 0.36, respectively) [71]. Radial as compared with femoral access was shown to have consistent benefit across the whole spectrum of patients with ACS, resulting in upgrading of the recommendation to a class I indication in the 2017, 2020, and 2023 ESC guidelines.

In Japan, the TEMPURA trial randomized patients with AMI undergoing primary PCI to transradial coronary intervention (TRI) group (*n* = 77) and transfemoral coronary intervention (TFI) group (*n* = 72) [72]. The success rate of reperfusion and the incidence of in-hospital MACE were similar in both groups (96.1% and 5.2% vs 97.1% and 8.3% in TRI and TFI groups, respectively). In a substudy of PRASFIT-ACS including ACS patients with prasugrel, rates of periprocedural bleeding, bleeding not related to CABG, and puncture site bleeding were consistently lower in the TRI group than in the TFI group [73]. More recently, in a report from the CREDO-Kyoto AMI registry was published [74]. A total of 3662 STEMI patients who had primary PCI by TRI (*N* = 471) or TFI (*N* = 3191) were analyzed. The prevalence of hemodynamically compromised patients (Killip II–IV) was significantly less in the TRI group than in the TFI group (19 vs 25%, *P* = 0.002). Cumulative 5-year incidences of death/MI/stroke, and major bleeding were not significantly different between the TRI and TFI groups (26.7 vs 25.9%, log-rank *P* = 0.91, and 11.3 vs 11.5%, log-rank *P* = 0.71, respectively). After adjustment for confounders, the risks of the TRI or TFI group were not significant for both death/MI/stroke [hazard ratio (HR) 1.15, 95% confidence interval (CI) 0.83–1.59, *P* = 0.41] and major bleeding (HR 1.29, 95% CI 0.77–2.15, *P* = 0.34), leading to the conclusion that clinical outcomes of a transradial approach were not different from those of a transfemoral approach in primary PCI for STEMI in the real-world practice.

### Recommendations

- Radial access is recommended over femoral access if performed by an experienced radial operator.

### Thrombus aspiration

While it has been well recognized that thrombus formation caused by plaque rupture, plaque erosion, and calcified nodules plays a crucial role in the mechanism of ACS, the reduction of thrombus burden can theoretically be effective therapy for AMI [75–79]. However, in the guidelines released by the European Society of Cardiology in 2023 on the management of patients with ACS, routine thrombus aspiration is not recommended (class III, Level A).

A pooled analysis of individual-patient data from three large randomized trials (TAPAS [Thrombus Aspiration During Percutaneous Coronary Intervention in Acute Myocardial Infarction], TASTE [Thrombus Aspiration in ST-Elevation Myocardial Infarction in Scandinavia], and TOTAL [Trial of Routine Aspiration Thrombectomy With PCI Versus PCI Alone in Patients With STEMI]) provided novel insights about thrombus aspiration for ST-elevation MI [80]. Despite including 18,306 patients, the study did not show a significant reduction in cardiovascular death when thrombus aspiration was compared with standard therapy. There were also no differences between thrombus aspiration and no thrombus aspiration with respect to stroke or transient ischemic attack, recurrent MI, stent thrombosis, heart failure, or target-vessel revascularization [81]. Although routine use of mechanical thrombus aspiration is no longer recommended, prior safety concerns regarding the risk of stroke could not be confirmed. However, because a trend toward reduced cardiovascular death and increased stroke or transient ischemic attack was found in the subgroup of patients with high thrombus burden, future studies may want to investigate improved thrombus aspiration technologies in this high-risk subgroup.

In contrast to the studies mentioned above, earlier studies had shown a potential benefit for thrombus aspiration in primary PCI [82, 83].

### Evidence from Japan

In the J-PCI registry 2022, thrombus aspiration was used in 43.0% of STEMI, 14.5% of NSTEMI, and 3.7% of UA (Table 2). The high utilization rate of thrombus aspiration in Japan can be attributed to the inability to use GP IIb/IIIa inhibitors as well as evidence from Japan which showed the benefit of thrombus aspiration.

In the VAMPIRE study, patients with STEMI were randomized to primary PCI with (*n* = 180) or without (*n* = 175) upfront thrombus aspiration [84]. There was a trend toward a lower incidence of slow or no reflow (primary endpoint defined as a Thrombolysis in Myocardial Infarction flow grade < 3) in patients treated with aspiration versus conventional primary PCI (12.4% vs 19.4%, *P* = 0.07). The rate of myocardial blush grade 3 was higher in the aspiration group (46.0% vs 20.5%, *P* < 0.001). Aspiration was most effective in patients presenting after 6 h of symptom onset (slow-flow rate: 8.1% vs 37.6%, *P* = 0.01). Patients presenting late after STEMI appear to benefit the most from thrombectomy.

In an observational study (*n* = 3913) by Nakatani et al. [85], thrombus aspiration was associated with a lower 30-day mortality rate in selected patients with high TIMI risk scores, an age > or = 70 years, diabetes mellitus, or stenting adjusted for baseline characteristics.

In the latest guidelines of Japanese Circulation Society, thrombus aspiration in primary PCI was recommended as a class IIa indication with level of evidence B. Accordingly, thrombus aspiration is performed frequently in primary PCI in Japan. A comparison of specifications of aspiration device is tabulated in Table 5. From a practical view point, aspiration performance, trackability, and pushability, are of importance when choosing an aspiration catheter [86].

**Table 5:** Thrombus aspiration catheters commercially available in Japan

Company	Product name	Guiding catheter compatibility	Guidewire compatibility (inch)	Catheter length (cm)	Wire lumen length (mm)	Distal outer diameter (mm)	Distal inner diameter (mm)	Proximal outer diameter (mm)	Proximal inner diameter (mm)	Length of hydrophilic coating (cm)	Shape of aspiration lumen	Stylet
Terumo	Eliminate + SL	6Fr	0.014	140	90	1.70	0.98	1.40	1.05	40	Circle	No
	Eliminate + XL	6Fr	0.014	140	90	1.75	1.10	1.40	1.15	40	Circle	Yes
		7Fr	0.014	140	90	1.98	1.30	1.60	1.35	40	Circle	Yes
Medtronic	Export Advance	6Fr	0.014	140	200	1.70	1.09	1.37	1.12	38	Circle	Yes
Kaneka	Thrombuster Pro SL	6Fr	0.014	140	200	1.36	1.00	1.36	1.00	30	Circle	No
		7Fr	0.014	140	200	1.56	1.25	1.56	1.25	30	Circle	No
	Thrombuster Pro GR	6Fr	0.014	140	120	1.36	1.10	1.36	1.16	30	Circle	Yes
		7Fr	0.014	140	120	1.56	1.32	1.56	1.36	30	Circle	Yes
Nipro	TVAC II	6Fr	0.014	140	240	1.77	0.95	1.40	0.95	24	Circle	Yes^a^
		7Fr	0.014	140	240	1.90	1.18	1.60	1.18	24	Circle	Yes^a^
	TVAC SOFT	6Fr	0.014	135	250	1.50	NA	1.30	NA	25	Crescent	No
		7Fr	0.014	135	250	1.50	NA	1.50	NA	25	Crescent	No
		8Fr	0.014	135	250	1.80	NA	1.80	NA	25	Crescent	No
Goodman	Rebirth III	6Fr	0.014	136	220	1.31 × 1.67	1.05	1.38	1.05	60	Circle	No
		7Fr	0.014	136	220	1.56 × 1.91	1.37	1.62	1.37	60	Circle	Yes
	Rebirth Pro 2	6Fr	0.014	136	220	1.35 × 1.62	1.09	1.38	1.11	60	Circle	Yes
		7Fr	0.014	136	220	1.60 × 1.90	1.34	1.58	1.25	60	Circle	Yes
Zeon	ZEEK IV	6Fr	0.014	135	170	1.30	1.11	1.37	1.11	30	Crescent	Yes

^a^There is TVAC II with or without stylet

Anzai et al. reported that thrombus aspiration facilitates direct stenting without increasing the cost of treatment [87]. Thrombus aspiration can be considered followed by direct stenting, which will be discussed later.

Inohara et al. investigated the use of thrombus aspiration and its clinical impact in patients with ACS who were registered in J-PCI registry between 2016 and 2018. The rates of thrombus aspiration use decreased slightly during the period. Thrombus aspiration was associated with more successful PCI (TIMI 3 and residual stenosis < 25%). After adjustment, thrombus aspiration was not associated with in-hospital death in STEMI patients; however, it was associated with increased in-hospital mortality in NSTEMI or UA patients [38].

### Recommendations

- Although in Japan, GP IIb/IIIa inhibitors cannot be used, so there are no studies comparing the effectiveness of thrombus aspiration with GP IIb/IIIa inhibitors as the control arm, thrombus aspiration should be considered for thrombotic lesions at primary PCI in STEMI or PCI in NSTE-ACS patients in the absence of GP IIb/IIIa inhibitors in Japan.

### Distal protection

The benefit of distal protection using a filter device or occlusion balloon has not been confirmed [88, 89]. However, the use of distal protection devices can be considered when the plaque burden is large and there is a high possibility of distal embolism or no reflow. In J-PCI registry 2022, distal protection devices were used in 5.2% of STEMI, 2.9% of NSTEMI, and 2.5% of UA (Table 4).

### Evidence from Japan

Isshiki et al. reported initial clinical experience with Filtrap™ distal protection filter [90]. Filtrap™ was successfully delivered and deployed distal to the lesion in 13 of 14 patients (93%). Embolic debris was entrapped in 8 (62%) of these cases. All patients were free from in-hospital events except for one patient with a large anterior AMI who received emergency surgery due to a free wall cardiac rupture. In the ASPARAGUS trial (*n* = 341), patients with AMI were randomized to either stenting with or without GuardWire Plus™ [91]. The rates of slow-flow and no-reflow immediately after PCI were 5.3 and 11.4% in the GuardWire Plus and control groups, respectively (*P* = 0.05). Blush score 3 acquisition rates immediately after PCI were 25.2 and 20.3% in the GuardWire Plus and control groups, respectively (*P* = 0.26), and the rates at 30 days after PCI were 42.9 and 30.4%, respectively (*P* = 0.035). In the CANARY pilot trial, near-infrared spectroscopy and intravascular ultrasound were performed at baseline, and lesions with a maximal lipid core burden index over any 4-mm length (maxLCBI_4mm_) ≥ 600 were randomized to PCI with versus without a distal protection filter [92]. Among 31 randomized lesions with maxLCBI_4mm_ ≥ 600, there was no difference in the rates of periprocedural MI with versus without the use of a distal protection filter (35.7% vs 23.5%, *P* = 0.69). More recently, the VAMPIRE 3 trial randomized 200 ACS patients who had attenuated plaque with a longitudinal length of ≥ 5 mm by pre-PCI intravascular ultrasound to either distal protection (DP) by filter or conventional treatment (CT) [93]. The primary endpoint of no-reflow phenomenon occurred in 26.5% of the DP group (*n* = 98) and 41.7% of the CT group (*n* = 96; *P* = 0.0261) and the corrected TIMI frame count after revascularization was significantly lower in the DP group (23 vs 30.5; *P* = 0.0003). In addition, the incidence of in-hospital adverse cardiac events was significantly lower in the DP group than in the CT group (0% vs 5.2%; *P* = 0.028). Future studies may further elucidate whether distal protection is beneficial in selected patients.

In contrast, distal embolic protection during PCI of saphenous vein grafts was confirmed in a multicenter randomized-controlled trial. In the SAFER randomized trial, a composite of death, myocardial infarction, emergency bypass, or target-lesion revascularization by 30 days was observed in 16.5% in the control group and 9.6% in the embolic protection device (*P* = 0.004) [94]. This 42% relative reduction in major adverse cardiac events was driven by myocardial infarction (8.6% vs 14.7%, *P* = 0.008) and “no-reflow” phenomenon (3% vs 9%, *P* = 0.02). Clinical benefit was seen even when platelet glycoprotein IIb/IIIa receptor blockers were administered (61% of patients), with composite end points occurring in 10.7% of protection device patients versus 19.4% of control patients (*P* = 0.008). This study demonstrated the importance of preventing distal embolization in saphenous vein grafts.

Currently available filter devices in Japan are tabulated in Table 6.

**Table 6:** Filter devices for distal protection commercially available in Japan

Company	Product name	Filter diameter at expansion (mm)	Guidewire compatibility (inch)	Length (cm)
Nipro	Filtrap	3.5	0.014	180
		5	0.014	180
		6.5	0.014	180
		6.5	0.014	300
Tri-Med	Parachute	5	0.014	190
		5	0.014	270
		6.5	0.014	190
		6.5	0.014	270
		8	0.014	270
		8	0.014	50
		8	0.014	190

### Recommendations

- Distal protection can be considered in selective cases when the plaque burden is large and there is a high possibility of distal embolism or no reflow or cases with myocardial infarction in saphenous vein grafts.

### Pharmacological intervention for no reflow

In 2023 ESC guidelines [3], using GP IIb/IIIa inhibitors as bailout therapy is considered as a class IIa indication in the event of angiographic evidence of a large thrombus, slow- or no-reflow, although this strategy has not been tested in a randomized trial.

### Evidence from Japan

Ishii et al. performed a randomized trial among 368 STEMI patients undergoing primary PCI [the nicorandil group (*n* = 185) or control group (*n* = 183)] [95]. They reported that intravenous 12 mg of nicorandil before primary PCI significantly improved ST-segment resolution and epicardial coronary flow, resulting in the prevention of cardiovascular events of long duration and deaths, compared to placebo group.

Miyazawa et al. studied the effect of nicorandil in STEMI, randomizing patients with STEMI to the nicorandil group (*n* = 35) or control group (*n* = 35) [96]. In the nicorandil group, 2 mg of nicorandil was injected directly into the infarct area prior to reperfusion by PCI. With nicorandil infusion, additional ST elevation without chest pain was observed for a few minutes in 94% of cases. However, no ventricular fibrillation or ventricular tachycardia occurred. TIMI grade 3 rates were significantly higher in the nicorandil group (40% vs 17%, *P* < 0.01). Rates of adverse events were similar; however, left-ventricular regional wall motion score significantly improved in the nicorandil group (*P* < 0.05). The effect of nicorandil was pronounced in patients without ischemic preconditioning.

Kobatake et al. compared the effects of nitroprusside (*n* = 25) with nicorandil (*n* = 24) on the slow/no-reflow phenomenon during primary PCI [97]. The degree of improvement in TIMI flow grade (post- minus pre-TIMI flow grade divided by pre-TIMI flow grade) and TIMI frame count (pre- minus post-TIMI frame count divided by pre-TIMI frame count) showed that nitroprusside was more effective than nicorandil (nitroprusside vs nicorandil: 0.88 ± 0.79, 0.37 ± 0.37, *P* = 0.008; 0.59 ± 0.23, 0.36 ± 0.27, *P* = 0.003, respectively). At 1 year, the rate of MACE was not significantly different (5/25 vs 9/24, *P* = 0.175).

Further studies are needed to determine optimal methods of administration and doses of nicorandil, because nicorandil has dose-dependent effects on coronary artery diameters and coronary blood flow.

More recently, a network meta-analysis was published comparing the effect of seven intracoronary agents (adenosine, anisodamine, diltiazem, nicorandil, nitroprusside, urapidil, and verapamil) on the no-reflow phenomenon in patients with STEMI undergoing primary PCI, including 41 randomized control trials with 4069 patients [98]. Anisodamine (α1 adrenergic receptor antagonist used in the treatment of acute circulatory shock in China) was associated with improved post-procedural TIMI flow grade, more occurrences of ST-segment resolution, and improvement in left-ventricular ejection fraction (LVEF). The cardioprotective effect of anisodamine conferred a MACE-free survival benefit. Additionally, nitroprusside was regarded as efficient in improving coronary flow and clinical outcomes. Compared with standard care, adenosine, nicorandil, and verapamil improved coronary flow but had no corresponding benefits on cardiac function and clinical outcomes.

Considering GP IIb/IIIa inhibitors and anisodamine are not available in Japan, the use of nicorandil or nitroprusside prior to reperfusion by primary PCI may be considered reasonable.

### Recommendations

- Intravenous nicorandil may be considered for STEMI patients before primary PCI within 12 h after symptom onset to prevent coronary microvascular impairment.
- Intracoronary injection of nicorandil can be considered to bail out in case of slow-flow or no-reflow.

### Direct stenting

Evidence in favor of direct stenting (stenting without predilation) in patients with STEMI comes from several studies [99]. Loubeyre et al. randomized 206 patients with STEMI to direct stenting or stent implantation after balloon predilation [100].The composite angiographic (corrected TIMI frame count, slow-flow/no-reflow or distal embolization) endpoint (11.7% vs 26.9%; *P* = 0.01) and ST-segment resolution (79.8% vs 61.9%; *P* = 0.01) were better among patients randomized to direct stenting than among those randomized to stent implantation after predilation [100]. In the Harmonizing Outcomes with Revascularization and Stents in Acute Myocardial Infarction (HORIZONS-AMI), direct stenting (*n* = 698) compared with the conventional stenting after predilation (*n* = 1830) was associated with better ST-segment resolution at 60 min after the procedure (median: 74.8% vs 68.9%; *P* = 0.01) and lower 1-year rates of all-cause mortality (1.6% vs 3.8%; *P* = 0.01) and stroke (0.3% vs 1.1%; *P* = 0.049) [101]. The EUROTRANSFER Registry including 1,419 patients showed that direct stenting (*n* = 276) was superior to stenting after predilation in terms of post-procedural TIMI flow grade of 3 (94.9% vs 91.5%; *P* = 0.02), no-reflow (1.4% vs 3.4%; *P* = 0.035), ST-segment resolution of > 50% (86.2% vs 76.3%; *P* = 0.016), and one-year mortality (2.9% vs 6.5%; *P* = 0.047 after adjustment for propensity score) [102]. Direct stenting may be advantageous over stenting after predilation in several aspects including the use of fewer and shorter stents, shorter fluoroscopy time, and less use of contrast media and reduced microvascular dysfunction/obstruction and no-reflow by reduced distal embolization. Potential disadvantages of direct stenting may include: failure to reach and/or to cross the lesion, stent loss, erroneous estimation of stent length, difficulty with stent positioning (especially in cases of persistent TIMI flow 0–1), under-expansion of the stent in an undilatable (i.e., calcified) lesion, and stent undersizing due to underestimation of vessel diameter because of reduced flow [103]. Notwithstanding these disadvantages, direct stenting is now considered as an acceptable alternative strategy as compared to the conventional stenting during primary PCI.

### Recommendations

- Direct stenting should be considered in primary PCI.

### Plain old balloon angioplasty

The clinical efficacy of balloon angioplasty for STEMI is limited due to the relatively high percentage of restenosis caused by elastic recoil and late negative remodeling [104]. Several studies showed that the need for repeat revascularization was significantly reduced by the use of coronary stents [105–107]. There is also Japanese evidence supporting this fact in patients with AMI [108, 109]. Nonetheless, stent implantation did not result in lower rates of recurrent MI or death, when compared with balloon angioplasty alone. Subsequently, numerous randomized trials demonstrated that a further reduction in target-lesion revascularization (TLR) could be achieved when using drug-eluting stents (DES) as opposed to bare-metal stents (BMS). Equivalent to studies comparing balloon angioplasty with stenting, though none of these studies demonstrated a reduction in recurrent MI or death [110–112]. An important limitation of stent usage is a persistent risk of stent thrombosis (ST) and/or in-stent restenosis even years after implantation, particularly in patient subsets as STEMI [113–118].

Considering stent implantation may even induce no-reflow and thereby expand infarct size [119–121], it may be reasonable to refrain from stenting if coronary flow is restored and no significant stenosis persists after thrombus aspiration and balloon dilatation. Indeed, recent studies have demonstrated that it is safe to defer stent implantation in the acute phase of STEMI [122, 123]. Considering the absence of superiority with respect to hard clinical end points and the potential short- and long-term disadvantages of stent implantation, angioplasty with a drug-coated balloon (DCB) without stenting may well serve as a therapeutic strategy of choice in STEMI.

In the INNOVATION study, 114 patients receiving primary PCI for STEMI were randomized into deferred stenting (DS) or immediate stenting (IS) [124]. In the DS group, the primary procedures included thrombus aspiration and balloon angioplasty and the second-stage stenting procedure was scheduled to be performed at 3–7 days after primary reperfusion procedure. DS did not significantly reduce infarct size (15.0% vs 19.4%; *P* = 0.112) and the incidence of microvascular obstruction (MVO; 42.6% vs 57.4%; *P* = 0.196), compared with IS. However, in anterior wall myocardial infarction, infarct size (16.1% vs 22.7%; *P* = 0.017) and the incidence of MVO (43.8% vs 70.3%; *P* = 0.047) were significantly reduced in the DS group.

### Recommendations

- Currently, a POBA-only strategy is not recommended over direct stenting.

### Stent

#### Drug-eluting stents

Some meta-analyses suggested the safety and efficacy of second-generation DES in STEMI patients. In a network meta-analysis of patients with STEMI undergoing primary PCI (12,453 patients from 22 trials) [125], cobalt–chromium everolimus-eluting stents (CoCr-EES) were associated with significantly lower rates of cardiac death or MI and stent thrombosis (ST) than BMS. CoCr-EES was also associated with significantly lower rates of 1-year ST than paclitaxel-eluting stents (PES). Sirolimus-eluting stents (SES) were also associated with significantly lower rates of 1-year cardiac death/myocardial infarction than BMS. CoCr-EES, PES, and SES, but not zotarolimus-eluting stents, had significantly lower rates of 1-year target-vessel revascularization (TVR) than BMS, with SES also showing lower rates of TVR than PES. Another network meta-analysis with longer follow-up data analyzed 12 trials with 9,673 patients [126]. Second-generation DES were associated with significantly lower incidence of definite or probable ST (OR 0.59, 95% CI 0.39–0.89), MI (OR 0.59, 95% CI 0.39–0.89), and TVR at 3 years (OR 0.50: 95% CI 0.31–0.81) compared with BMS. In addition, there was a significantly lower incidence of MACE with second-generation DES versus BMS (OR 0.54, 95% CI 0.34–0.74) at 3 years. In a patient-level network meta-analysis in patients with STEMI undergoing primary PCI with a median follow-up of 3 years (10,979 patients from 15 trials) [127], DES were superior to BMS with respect to cardiac death, reinfarction, or target-lesion revascularization (TLR), and definite or probable stent thrombosis. Although second-generation DES did not significantly reduce cardiac death, reinfarction, or TLR, compared to first-generation DES (HR 0.98, 95% CI 0.79–1.21), second-generation DES were better than first-generation DES in the reduction of definite or probable stent thrombosis (HR 0.56, 95% CI 0.36–0.88).

In terms of long follow-up, recently, the EXAMINATION-EXTEND (10-Year Follow-Up of the EXAMINATION Trial) study demonstrated the superiority of CoCr-EES (*N* = 751) in combined patient- and device-oriented composite endpoints, compared with BMS (*N* = 747), in patients with STEMI (patient-oriented composite endpoint: 32.4% vs 38.0%, HR 0.81, 95% CI 0.68–0.96, *P* = 0.013; device-oriented composite endpoint: 13.6% vs 18.4%, HR 0.72, 95% CI 0.55–0.93, *P* = 0.012, respectively) [128]. These results were driven mainly by TLR (5.7% vs 8.8%; *P* = 0.018). The rate of definite stent thrombosis was similar in both groups (2.2% vs 2.5%; *P* = 0.590). No differences were found between the groups in terms of target-lesion revascularization (1.4% vs 1.3%; *P* = 0.963) and definite or probable stent thrombosis (0.6% vs 0.4%; *P* = 0.703) between 5 and 10 years.

#### Ultrathin strut DES

The non-inferiority of a new-generation ultrathin strut DES with biodegradable polymer, Orsiro, compared with Xience with respect to target-lesion failure at 1 year was demonstrated in the BIOSCIENCE trial [129]. The subgroup analysis showed favorable outcomes of Orsiro in STEMI patients. Following this study, BIOSTEMI trial [130] was conducted enrolling 1,300 STEMI patients. The statistical analysis of BIOSTEMI trial incorporated the STEMI population in the BIOSCIENCE trial. The primary endpoint of target-lesion failure (TLF: cardiac death, target-vessel MI, and clinically indicated TLR) at 1 year was 4% with Orsiro and 6% with Xience (difference -1.6%; rate ratio 0.59, 95% Bayesian credibility interval 0.37–0.94; posterior probability of superiority 0.986), and Orsiro was superior to Xience. The difference was mainly driven by the lower rate of ischemia-driven TLR in Orsiro. The superiority of Orsiro was also reported at 5-year follow-up [131].

Overall, use of new-generation DES is encouraged, although the clinical benefit of ultrathin strut DES should be further investigated.

#### Drug-coated stents

The LEADERS FREE (Prospective Randomized Comparison of the BioFreedom Biolimus A9 Drug-Coated Stent versus the Gazelle Bare-Metal Stent in Patients at High Bleeding Risk) study compared the polymer-free biolimus-eluting Biofreedom stent with a bare-metal stent (BMS) in a cohort (*N* = 2466) at high risk of bleeding [132]. In a subgroup analysis of 659 ACS patients, treatment with the BioFreedom stent remained more effective (clinically driven target-lesion revascularization 3.9% vs 9.0%, *P* = 0.009) and safer (cumulative incidence of cardiac death, MI, or definite or probable stent thrombosis 9.3% vs 18.5%, *P* = 0.001), driven by significantly lower rates of cardiac mortality (3.4% vs 6.9%, *P* = 0.049) and MI (6.9% vs 13.8%, *P* = 0.005) [133].

The SORT OUT IX trial was an RCT which assessed the non-inferiority for MACE of BioFreedom compared with Orsiro in all-comers population (*n* = 3151). BioFreedom failed to show the non-inferiority at 1 year (5.0% vs 3.7%, *P*_non-inferiority_ = 0.14). In the subgroup analysis, Orsiro was significantly favored in ACS patients [134].

### Evidence from Japan

There are scarce randomized studies comparing stents in Japan. Sawada et al. randomized patients with STEMI to receive EES (*n* = 23) or SES (*n* = 12) and compared arterial healing by OCT [135]. Both the EES and SES showed an excellent suppression of neointimal proliferation in the culprit lesion. The frequency of uncovered and malapposed struts of EES was significantly lower than that of SES (2.7% vs 15.7%, *P* < 0.0001, 0.7% vs 2.3%, *P* < 0.0001, respectively). EES may promote better arterial healing response than SES in patients with STEMI.

### Recommendations

- Stenting with recent generation DES is recommended over BMS for PCI in ACS.

#### Drug-coated balloon

The evidence regarding safety and efficacy of drug-coated balloons (DCB) in ACS patients is limited [136, 137].

PAPPA was a pilot study which investigated the safety and efficacy of paclitaxel-coated balloons in 100 STEMI patients. [138] Additional stenting was required in 41% patients and the primary composite endpoint of cardiac death, recurrent MI in the target vessel and TLR occurred in 5% patients. The REVELATION trial randomized 120 STEMI patients either to paclitaxel-coated balloon or DES and evaluated FFR at 9 months. [139] Bailout stenting was required in 18% of DCB patients. The FFR value at 9 months after DCB treatment was non-inferior to DES (0.92 ± 0.05 vs 0.91 ± 0.06, *P* = 0.27); however, the dropout rate of this study was high. PEPCAD NSTEMI trial randomized 210 NSTEMI patients either to paclitaxel-coated balloon or BMS/DES, and the DCB treatment was non-inferior to BMS/DES in terms of target-lesion failure (composite of cardiac or unknown death, MI and TLR) at 9 months (3.8% vs 6.6%, *P* = 0.53) [140]. In the DCB group, 15% of patients required additional stent implantation.

Recently, the results of REC-CAGEFREE II trial which was conducted in China were presented at EuroPCR 2024. This was a prospective open-label non-inferiority trial which compared step-wise de-escalation of DAPT with standard antiplatelet therapy in patients with ACS (STEMI, NSTEMI, or UA) treated with paclitaxel-coated balloons. In the de-escalation group, patients received aspirin plus ticagrelor for 1 month, followed by 5 months of ticagrelor monotherapy, and then 6 months of aspirin monotherapy. In the standard group, patients received 12 months of DAPT (aspirin + ticagrelor). The primary endpoint was non-inferiority for net adverse clinical events (NACE; all-cause death, stroke, MI, revascularization, and BARC type 3 or 5 bleeding) assessed at 12 months. The de-escalation group met the criteria for non-inferiority (9.0% vs 8.7%; absolute difference 0.31%; upper boundary of the one-sided 95% CI 2.43%; *P*_non-inferiority_ = 0.013). In the win ratio analysis, which evaluated the components of the primary composite endpoint in a hierarchical way, the de-escalation group had significantly more wins than the standard group (*P* = 0.004).

There are several concerns for using DCB in ACS patients. First, thrombus at the culprit lesion may prevent delivery of the drug to the vessel wall. A second concern is acute vessel occlusion which was frequently observed in the era of POBA. One patient (1%) in PAPPA study [138], one patient (0.8%) in REVALATION study [139], and no patients in PEPCAD NSTEMI [140] experienced acute vessel occlusion. Dedicated balloon sizing based on the IVUS/OCT and careful interpretation of both angiography and IVUS/OCT may reduce the risk of complications after DCB treatment. Third, because the type, dosage, and formulation of drug vary among the different DCBs, there might be no class effect of DCB [137]. Thus, the results from one DCB may not be applicable to another DCB.

These results may support the use of DCB in ACS patients, but obviously further studies are needed.

### Recommendations

- DCB can be considered for PCI in ACS patients if implanting DES is not feasible.

### Intracoronary imaging (IVUS/OCT/OFDI)

#### Intracoronary imaging for ACS

In the 2023 ESC guidelines for ACS, the use of intracoronary imaging is newly recommended as follows; “Intravascular imaging should be considered to guide PCI (Class IIa, Level A)” and “Intravascular imaging (preferably optical coherence tomography) may be considered in patients with ambiguous culprit lesions (Class IIb, Level C)”. Recent meta-analyses report that intracoronary imaging-guided PCI is associated with better outcomes compared with angiography-guided PCI [141–143]. In Japan, intracoronary imaging is reimbursed, resulting in a high utilization rate of 93% in the STOPDAPT-3 trial and 94% in PENDULUM registry [59, 144].

#### IVUS for ACS

In the IVUS-XPL trial [145, 146], 1400 patients (49% with ACS) with long lesions were randomized to IVUS versus angiographic guidance. IVUS guidance was associated with a lower MACE rate of 2.9% vs 5.8% at 1 year (*P* = 0.007), and subgroup analysis showed favorable outcomes of IVUS guidance in patients with ACS (HR 0.35, 95% CI 0.16–0.75). Five-year follow-up data confirmed the benefit of IVUS guidance in reducing MACE (5.6% vs 10.7%, *P* = 0.001).

The ULTIMATE trial [147] randomized 1448 patients (78.5% with ACS) either to IVUS or angiographic guidance. IVUS guidance was associated with a lower target-vessel failure rate of 2.9% vs 5.4% (HR 0.53, *P* = 0.019) at 1 year. Sub-group analysis showed that IVUS guidance reduced the rate of TVF in patients with ACS (HR 0.56, 95% CI 0.32–0.99). The IVUS guidance group maintained favorable results at 3 years with the TVF rate of 6.6% in IVUS guidance and 10.7% in angiography guidance (*P* = 0.01), and the stent thrombosis rate of 0.1% in IVUS guidance and 1.1% in angiography guidance (*P* = 0.02) [148].

Recently, the results of IVUS-ACS trial were reported [149]. This was a two-stage randomized trial, which randomized ACS (STEMI, NSTEMI or UA) patients to IVUS-guided or angiography-guided groups, followed by second randomization either to 1-month or 12-month DAPT at 1 month follow-up (ULTIMATE-DAPT trial [150]). 1753 patients were randomized to IVUS- or an angiography-guided group. IVUS guidance had a significantly lower rate of the target-vessel failure (a composite of cardiac death, target-vessel MI, or clinically driven target-vessel revascularization) at 1 year (4.0% vs 7.3%; hazard ratio 0.55; 95% CI 0.41–0.74; *P* = 0.0001). The incidence of target-vessel MI, non-procedural MI, and clinically driven target-vessel revascularization were significantly lower in the IVUS-guided group than in the angiography-guided group.

#### OCT for ACS

In CLI-OPCI observational study (*n* = 670), OCT guidance was associated with a significantly lower risk of cardiac death or MI as compared to angiographic only guidance at 1 year (adjusted OR = 0.49 [0.25–0.96], *P* = 0.037) [151].

Several randomized-controlled trials have reported mechanistic or physiological benefits of OCT-guided PCI compared with angiography-guided PCI. OCTACS study randomized NSTEMI patients either to OCT-guided or angiography-guided PCI and conducted OCT follow-up at 6 months. OCT guidance was associated with a lower rate of malapposed stent struts in the post-procedural OCT assessment (3.4% vs 7.8%, *P* < 0.01), and a lower proportion of uncovered struts at 6 months (4.3% vs 9.0%, *P* < 0.01) [152]. The DOCTORS study randomized 240 NSTE-ACS patients either to OCT-guided or angiography-guided PCI, showing a significantly higher post-PCI fractional flow reserve (FFR) in the OCT-guided group (0.94 ± 0.04 vs 0.92 ± 0.05, *P* = 0.005) [153]. Kala et al. reported sub-analysis of the ROBUST trial, which randomized STEMI patients to OCT-guided vs angiography-guided PCI and conducted OCT follow-up at 9 months. OCT-guided PCI significantly reduced the in-segment area stenosis at 9 months [154]. EROSION III study, randomizing 246 STEMI patients with early infarct artery patency either to OCT guidance or angiography guidance. OCT guidance significantly reduced the rate of stent implantation (43.8% vs 58.8%, *P* = 0.024), and the residual angiographic diameter stenosis (8.7 ± 3.7% vs 11.8 ± 4.6%, *P* < 0.001) [155].

Recently, 2 large RCTs investigating the effect of OCT on clinical endpoint were published. ILUMIEN IV trial randomized 2487 patients (57% with ACS) with high-risk features either to OCT-guided or angiography-guided PCI. OCT guidance demonstrated a larger minimum stent area (5.72 ± 2.04 vs 5.36 ± 1.87 mm^2^, *P* < 0.001); however, there was no significant difference in the rate of target-vessel failure at 2 years (7.4% vs 8.2%, HR 0.90, 95% CI 0.67–1.19, *P* = 0.45). The rate of stent thrombosis was lower in the OCT-guided group (0.5% vs 1.4%, HR 0.36, 95% CI 0.14–0.91, *P* = 0.02) [156]. OCTOBER was a RCT which compared OCT guidance and angiography guidance in 1201 patients (46% with ACS) with a complex bifurcation lesion. OCT guidance significantly reduced the rate of MACE at 2 years (10.1% vs 14.1%, HR 0.70, 95% CI 0.50–0.98, *P* = 0.035) [157].

#### IVUS or OCT vs angiography

RENOVATE-COMPLEX-PCI randomized 1639 patients (51% with ACS) either to intracoronary imaging (IVUS or OCT) guided- or angiography-guided PCI. IVUS and OCT were used in 74% and 26%, respectively, in the intracoronary imaging-guided group. During a median follow-up period of 2.1 years, intracoronary imaging-guided PCI reduced the rate of target-vessel failure (7.7% vs 12.3%, HR 0.64, 95% CI 0.45–0.89, *P* = 0.008). In the subgroup analysis, both IVUS and OCT were associated with a lower rate of target-vessel failure [158].

#### IVUS vs OCT vs angiography

ILUMIEN III was an RCT which compared post-PCI minimum stent area measured by OCT among IVUS-, OCT- and angiography-guided PCI in 450 patients with 36% ACS. ILUMIEN III used a dedicated OCT stent optimization strategy using external elastic membrane diameters. OCT-guided PCI was non-inferior to IVUS- or angiography-guided PCI in terms of minimum stent area. In addition, there were no significant difference in the rates of target-vessel failure and MACE at 1 year [159]. The iSIGHT trial compared the stent expansion among IVUS-, OCT- and angiography-guided PCI in 150 patients with 59% ACS, using a dedicated OCT strategy based on external elastic membrane. The OCT guidance was non-inferior to IVUS guidance, and superior to angiography guidance [160].

#### Meta-analysis

Two network meta-analyses comparing intravascular imaging-guided PCI vs angiography-guided PCI have been published this year [142, 143]. Both studies included more than 20 RCTs and 15,000 patients. Despite some differences in the results of the two meta-analyses, intravascular imaging (IVUS or OCT)-guided PCI was associated with reduced risks of cardiac death, all-cause death, target-vessel MI, target-lesion revascularization, and stent thrombosis when compared to angiography-guided PCI. When intravascular imaging was divided into IVUS and OCT, IVUS reduced the risks of cardiac death, target-lesion revascularization, and target-vessel revascularization, whereas OCT was associated with a lower risk of stent thrombosis compared with angiography guidance. Different inclusion/exclusion criteria among studies require a cautious approach to interpreting results; however, there is no doubt that both IVUS and OCT offer detailed observation of coronary lesions. Considering that intravascular imaging is reimbursed by insurance in Japan, the use of intravascular imaging during PCI is recommended.

#### Identification of culprit lesion

Optical coherence tomography (OCT), Optical frequency domain imaging (OFDI), and Intravascular ultrasound (IVUS) detect plaque ruptures in about half of ST-elevation myocardial infarction. However, the superior resolution and obligatory flushing with OCT sharply outlines the rupture cavity and residual fibrous cap fragment to optimize ruptured plaque identification. de Feyter and Ozaki previously demonstrated plaque rupture and thrombus were more frequently found in ACS than those with stable angina by angioscopy, while IVUS failed to discriminate unstable from stable plaque [161]. More recently, Kubo et al. reported, when compared with the gold standard of angioscopy, OCT can identify thrombus better than IVUS and differentiate between red and white thrombus, although red thrombus can shadow and obscure underlying plaque morphology [79].

While pathological studies report that plaque erosion plays a role in ACS, there was no clear OCT definition of plaque erosion previously. While Ozaki and his colleagues proposed that OCT-derived intact fibrous cap (IFC-ACS) can be plaque erosion for the first time, contrary to ruptured fibrous cap (RFC-ACS), distinct culprit lesion characteristics associated with IFC-ACS mechanisms are not identified by CT angiography or IVUS [78]. OCT has been used to monitor changes in thrombus burden when lesions are treated with thrombus aspiration or with pharmacotherapy [162, 163]. Prati et al. demonstrated in the CLIMA study that the simultaneous presence of four high-risk OCT plaque features [MLA < 3.5 mm^2^, FCT < 75 μm, lipid arc circumferential extension > 180°, OCT-defined macrophages] was found to be associated with a higher risk of major coronary events in 1003 patients undergoing OCT evaluation of the untreated proximal LAD [164].

In addition, combined IVUS and Near-Infrared Spectroscopy (NIRS) imaging, in particular where an increased plaque burden and lipid component are present, is able to differentiate culprit lesions from non-culprit lesions with a high accuracy in STEMI [165, 166] and NSTEMI [167].

#### Distal embolization or periprocedural myocardial infarction during stent implantation

Thin-cap fibrous atheroma (TCFA) not only causes plaque rupture and thrombosis but also contributes to myonecrosis during stenting. Findings associated with peri-myocardial infarction are greyscale IVUS-attenuated plaques, especially when the amount of attenuated plaque is large and begins closer to the lumen than to the adventitia; when large virtual histology-IVUS necrotic core or a virtual histology-thin-cap fibroatheroma or similar findings with integrated backscatter-IVUS (lipid) or iMap (necrotic core) are present; when an OCT-TCFA is present; when large lipid-rich plaques are detected by OCT or NIRS; or when plaque rupture is detected by IVUS or OCT [168, 169]. Furthermore, Ozaki and his colleagues reported that IB-IVUS-identified TCFA as well as OCT-verified TCFA were significant independent predictors of periprocedural myocardial infarction (PMI) after PCI [169]. However, the positive predictive value is poor and one trial [92] did not show superiority of distal protection when treating lipid-rich plaques. Conversely, the absence of these findings indicates a low probability of a peri-myocardial infarction with a high negative predictive value.

#### Postprocedural IVUS/OCT

Postprocedural IVUS/OCT is used to evaluate stent under-expansion, malapposition, tissue protrusion, dissection, geographic miss, and thrombus. In the expert consensus document of the EAPCI [170], a relative stent expansion of > 80% (minimal stent area [MSA] divided by average reference lumen area), and an MSA of > 5.5 mm^2^ by IVUS and > 4.5 mm^2^ by OCT in non-left main lesions are recommended.

Prati and his colleagues reported that a total of 1,002 lesions (832 patients) were assessed. Appropriate OCT assessment was obtained in 98.2% of cases and revealed suboptimal stent implantation in 31.0% of lesions, with increased incidence in patients experiencing major adverse cardiac events (MACE) during follow-up (59.2% vs 26.9%; *P* < 0.001). They concluded that suboptimal stent deployment defined according to specific quantitative OCT criteria was associated with an increased risk of MACE during follow-up in CLI-OPCI II study [171]. Prati and his coworkers also indicated that in ACS patients undergoing PCI, a composite of OCT-defined suboptimal stent implantation characteristics at the culprit lesion and residual intrastent plaque/thrombus protrusion was associated with adverse outcome in the CLI-OPCI ACS substudy [172].

OCT-guided PCI is non-inferior to IVUS-guided PCI in terms of stent expansion in the ILUMIEN III trial [159] and clinical outcome in the OPINION trial [173] from Japan. In general, a small edge dissection found on OCT which is undetected on angiography most likely does not have a clinical impact [174–177]. However, the following factors need to be considered: longitudinal and circumferential extension of dissection, and the depth of dissection (intima, media or even adventitia). In ILUMIEN III [159], edge dissections were categorized as major if they constituted ≥ 60° of the circumference of the vessel at the site of dissection and/or were ≥ 3 mm in length. In that trial, when the intra-dissection lumen area is < 90% of the respective reference area, additional stent implantation was considered. In CLI-OPCI-II trial [171], dissection was defined on OCT as a linear rim of tissue with a width of ≥ 0.2 mm and a clear separation from the vessel wall or underlying plaque. In this retrospective multicenter registry, acute dissection in the distal stent edge was an independent predictor for major adverse cardiac events.

If the malapposition distance from the endoluminal lining of strut to the vessel wall is < 250 µm, such struts likely come into contact with vessel wall at follow-up. Therefore, such small malappositions may be less clinically relevant [178, 179]. The clinical relevance of acute malapposition on stent failure is not yet fully established [171, 180–182]. Ozaki et al. reported that acute strut malapposition could persist (persistent malapposition; 4.67%) or resolve at follow-up (resolved/healed malapposition; 2.48%), whereas strut malapposition could also develop during follow-up (late acquired malapposition; 0.37%) [183]. The temporal evolution and disappearance of malapposition makes the investigation of the clinical relevance of strut malapposition more complicated.

#### Vulnerable plaque

Vulnerable plaque refers to high-risk plaques that have the potential to cause ACS in the future [184, 185]. The features of vulnerable plaque include TCFA, high plaque burden, low MLA, larger lipid core burden index, etc. Vulnerable plaque lesions often do not appear severe on angiography or upon hemodynamic assessment. Whether to perform preventive PCI to non-flow limiting vulnerable plaque lesions is controversial.

In the COMBINE FFR-OCT prospective, double-blind, natural history study, the impact of TCFA with FFR-negative lesions was investigated in patients with diabetes mellitus. The primary endpoint (a composite of cardiac mortality, target-vessel myocardial infarction, clinically driven target-lesion revascularization or unstable angina requiring hospitalization) occurred more frequently in TCFA-positive than in TCFA-negative patients at 18 months (13.3% vs 3.1%; hazard ratio 4.65; 95% CI 1.99–10.89; *P* < 0.001) [186].

PECTUS-obs observational study investigated the impact of FFR-negative high-risk plaques in patients with MI (STEMI and NSTEMI). OCT was performed on FFR-negative non-culprit lesions and patients were followed up. At 2 years, the primary endpoint (a composite of all-cause death, nonfatal MI, or unplanned revascularization) occurred more frequently in patients with a high-risk plaque than those without (15.4% vs 8.3%; hazard ratio 1.93[95% CI 1.08–3.47]; *P* = 0.02) [187].

The PREVENT trial randomized 1606 patients with non-flow-limiting vulnerable plaques either to PCI plus optimal medical therapy (OMT) or OMT alone. The PCI group had a significantly lower rate of the primary endpoint (a composite of death from cardiac causes, target-vessel myocardial infarction, ischemia-driven target-vessel revascularization, or hospitalization for unstable or progressive angina) at 2 years compared to OMT group (0.4% vs 3.4%; absolute difference −3.0% [95% CI −4.4 to −1.8]; *P* = 0.0003) [188].

The PREVENT trial showed the benefit of preventive PCI to non-obstructive vulnerable plaques; however, we still need to investigate the clinical impact including the cost-effectiveness.

### Recommendations

- IVUS or OCT should be used to guide optimal PCI.
- A relative stent expansion of > 80% (MSA divided by average reference lumen area), and an MSA of > 5.5 mm^2^ by IVUS and > 4.5 mm^2^ by OCT in non-left main lesions should be achieved.
- Acute incomplete stent apposition with a distance of ≤ 250 micron is likely to be resolved at follow-up. Additional post-dilatation is considered when malapposition distance is > 250 micron.
- Most edge dissection detected on OCT is clinically silent, whereas additional stenting may be performed if the width of distal edge dissection is ≥ 200 micron [171].
- IVUS/OCT/OFDI should be considered to detect stent-related mechanical problems.
- Postprocedural OCT/OFDI assessment includes the presence of dissection, degree of incomplete stent apposition, and presence of thrombus protrusion and may contribute to reducing MACE in long-term follow-up.
- Intracoronary imaging in non-target vessels should be considered to detect vulnerable plaques without physiologically significant stenosis which are prone to cause thrombotic events in the future. The pre-emptive stent could be considered to seal vulnerable plaque, taking into consideration procedural risk, ischemic and thrombotic risk, and patient preference.

### Intravascular physiology for the infarct-related artery

Intravascular physiology should not be used for the decision-making during the acute phase of AMI whether to perform or defer PCI for the infarct-related artery because the infarct-related artery is affected by microvascular obstruction [3]. As a post-PCI assessment in ACS patients, the index of microvascular resistance (IMR) is reportedly associated with infarct size and clinical outcomes [189, 190].

Physiological assessment of non-infarct-related artery in ACS patients with multivessel disease is described in the section: “Multivessel disease and treatment of non-infarct-related artery”.

### Mechanical circulatory support

#### IABP

Intra-aortic balloon pump (IABP) counterpulsation is the most widely used mechanical circulatory support (MCS) for the treatment of cardiogenic shock, based on the beneficial effect of aortic diastolic inflation and rapid systolic deflation, improving myocardial and peripheral perfusion and reducing afterload and myocardial oxygen consumption. However, IABP did not improve outcomes in patients with STEMI and cardiogenic shock without mechanical complications [191, 192], nor does it significantly limit infarct size in those with potentially large anterior MIs [193]. The latest ESC guidelines as well as JCS guidelines no longer recommend routine use of IABP in cardiogenic shock except selected patients (i.e., severe mitral insufficiency or ventricular septal defect) [194].

#### Impella

Impella is an intra-cardiac axial flow left-ventricular assist device which can be inserted via femoral or axillary arteries. Unlike VA-ECMO, Impella does not increase left-ventricular afterload. In Japan, it was approved for clinical use in 2017, and its usage has been increasing year by year [195, 196]. However, evidence regarding its benefits in ACS patients is limited. The ISAR-SHOCK trial investigated the hemodynamic impact of Impella LP 2.5 compared with IABP in 25 AMI-cardiogenic shock (AMI-CS) patients. Impella significantly increased the cardiac index at 30 min after implantation relative to IABP; however, mortality at 30 days was similar between the two groups [197]. Basir et al. reported the efficacy of early use of Impella in 406 patients with AMI-CS, reporting the survival rate after procedure, discharge, 30 days and 1 year as 99%, 71%, 68%, and 53%, respectively [198]. IMPRESS trial randomized 48 AMI-CS patients either to Impella CP percutaneous circulatory support device or IABP and did not find any benefit of Impella CP compared with IABP [199]. Schrage et al. retrospectively compared 30-day mortality of AMI-CS patients treated with Impella 2.5/CP with matched population from the IABP-SHOCK trial. Impella did not reduce 30-day mortality, but bleeding and vascular complications occurred more frequently in Impella [200].

Recently, the results of the DanGer Shock randomized trial were reported. 380 patients with STEMI and CS were assigned either to Impella CP plus standard care or standard care alone [27]. The primary endpoint, which was death from any cause at 180 days, less frequently occurred in Impella group compared with standard care group (45.8% vs 58.5%; hazard ratio 0.74; 95% CI 0.55–0.99; *P* = 0.04). However, the incidence of a composite safety endpoint (severe bleeding, limb ischemia, hemolysis, device failure, or worsening aortic regurgitation) was higher in the Impella group than in the standard group (24.0% vs 6.2%; relative risk 4.74; 95% CI 2.36–9.55).

There are several ongoing RCTs. The STEMI-DTU trial (NCT03947619) will compare primary left-ventricular unloading by Impella and a 30-min delay to reperfusion vs current standard of care in reducing infarct size and heart failure-related clinical events in patients presenting with anterior STEMI [201]. ULYSS (NCT05366452) and RECOVER IV (NCT05506449) trials will investigate whether Impella placement followed by PCI improves clinical outcomes compared to standard PCI in AMI-CS patients [202]. These trials will validate the safety and efficacy of Impella use prior to PCI in patients with AMI-CS.

#### ECMO

Venoarterial extracorporeal membrane oxygenation (VA-ECMO) is often used in a combination with IABP to reduce the afterload increased by the retrograde flow. In a retrospective cohort study using propensity score matching in the Japanese Diagnosis Procedure Combination national inpatient database [203], all-cause 28-day mortality and in-hospital mortality were significantly lower in the IABP combined with VA-ECMO group than the VA-ECMO-alone group (48.4% vs 58.2%; *P* = 0.001 and 55.9% vs 64.5%; *P* = 0.004, respectively). The proportion of patients weaned from VA-ECMO was significantly higher in the IABP combined with VA-ECMO group than in the VA-ECMO-alone group (82.6% vs 73.4%; *P* < 0.001).

#### Evidence from Japan

In Japan, Impella became available for clinical use in 2017. Nishimoto et al. reported the annual trends of mechanical circulatory support (MCS) use in Japan from 2010 to 2020 using the Japanese Diagnosis Procedure Combination database [195]. In the patients with cardiogenic shock (CS) requiring MCS, the proportion of the standalone IABP use significantly decreased from 80.5% in 2010 to 65.3% in 2020 (*P* for trend < 0.001), whereas the standalone Impella use significantly increased from 0.0% to 5.0% and ECMO use (regardless of whether combined with IABP or Impella) from 19.5% to 29.6% (*P* for trend < 0.001 for both). Nishimoto et al. also reported the trends and outcomes of ACS-CS patients undergoing PCI under MCS from J-PCI registry [196]. There were decreasing trends in IABP alone and VA-ECMO use, but an increasing trend in Impella use was found between 2019 and 2021. Although there was no significant improvement in in-hospital mortality in ACS-CS patients undergoing PCI under MCS after adjustment for confounders, a decreasing trend was observed. Saito et al. compared in-hospital outcomes between CS patients with and without AMI from the Japan Registry for Percutaneous Ventricular Assist Device [204]. AMI accounted for two-thirds of CS causes and the rates of in-hospital mortality and complications were similar between the two groups.

In other countries, mechanical LV assist devices (LVADs), including percutaneous short-term mechanical circulatory support devices (i.e., intra-cardiac axial flow pumps and arterial-venous extracorporeal membrane oxygenation) have been used in patients not responding to standard therapy, including inotropes, fluids, and IABP, but evidence regarding their benefits is still limited [205]. Therefore, short-term (MCS) may be considered as a rescue therapy to stabilize patients and preserve organ perfusion (oxygenation) as a bridge to recovery of myocardial function, cardiac transplantation, or even LV assist device destination therapy on an individual basis [206, 207].

A structured approach to determine the best adjunctive (MCS) device requires understanding the mechanisms, technical requirements, and hemodynamic responses of each device [208] (Table 7). Device escalation is often required if the initial support device (usually IABP) does not improve hemodynamics and end-organ perfusion. Venoarterial extracorporeal membrane oxygenation (VA-ECMO) is often used in a combination with IABP to reduce the afterload increased by the retrograde flow. In a retrospective cohort study using propensity score matching in the Japanese Diagnosis Procedure Combination national inpatient database [203], all-cause 28-day mortality and in-hospital mortality were significantly lower in the IABP combined with VA-ECMO group than the VA-ECMO-alone group (48.4% vs 58.2%; *P* = 0.001 and 55.9% vs 64.5%; *P* = 0.004, respectively). The proportion of patients weaned from VA-ECMO was significantly higher in the IABP combined with VA-ECMO group than in the VA-ECMO-alone group (82.6% vs 73.4%; *P* < 0.001).

**Table 7:** Comparison of mechanical circulatory support system

	IABP	IMPELLA	VA-ECMO
Cardiac flow	0.3–0.5 L/min	1–5 L/min (Impella 2.5, Impella CP, Impella 5)	3–7 L/min
Mechanism	Aorta	LV → Ao	RA → Ao
Maximum implant days	Weeks	7 days	Weeks
Sheath size	7–8 Fr	13–14 Fr Impella 5.0—21 Fr	14–16 Fr Arterial 18–21 Fr Venous
Femoral artery size	> 4 mm	Impella 2.5 & CP: 5–5.5 mm Impella 5: 8 mm	8 mm
Cardiac synchrony or stable rhythm	Yes	No	No
Afterload	↓	↓	↑↑↑
Mean arterial pressure	↑	↑↑	↑↑
LVEDP	↓	↓↓	⟷
PCWP	↓	↓↓	⟷
LV preload	–	↓↓	↓
Coronary perfusion	↑	↑	–
Myocardial oxygen demand	↓	↓↓	⟷

Modified from [208]

*Ao* aorta; *IABP* intra-aortic balloon pump; *LA* left atrium; *LV* left ventricle; *LVEDP* left-ventricular end-diastolic pressure; *RA* right atrium; *PCWP* pulmonary capillary wedge pressure; *VA-ECMO* venoarterial extracorporeal membrane oxygenation

There have been several clinical reports suggesting the combined use of Impella with IABP [209, 210]. However, this combination may decrease Impella forward flow during diastole due to diastolic pressure augmentation from the IABP [211].

The latest guidelines for ACS from Japanese Circulation Society recommend IABP use for the patients with mechanical complications as class I, however, do not recommend routine IABP use (class III) [194], considering that percutaneous LVADs are not broadly available in Japan.

### Recommendations

- Routine intra-aortic balloon pumping is not recommended.
- Intra-aortic balloon pumping should be considered in patients with hemodynamic instability/cardiogenic shock due to mechanical complications.
- In patients presenting refractory shock, short-term mechanical support (Impella or ECMO) may be considered in selected institutes.

### DAPT after PCI

#### Risk stratification for bleeding

The PRECISE-DAPT score (age, creatinine clearance, hemoglobin, white-blood-cell count, and previous spontaneous bleeding) was derived from 14,963 patients treated with different durations of DAPT (mainly aspirin and clopidogrel) after coronary stenting and showed a c-index for out-of-hospital TIMI major or minor bleeding of 0.73 (95% CI 0.61–0.85) [212]. A longer DAPT duration significantly increased bleeding in patients at high risk (score ~ 25) but did not in those with lower bleeding risk profiles, and exerted a significant ischemic benefit only in this latter group. As stated in the new ESC/EACTS Consensus document on DAPT, the use of risk scores such as PRECISE-DAPT designed to evaluate the benefits and risks of different DAPT durations “may be considered” to support decision-making [213].

Yoshikawa et al. reported that, in a pooled cohort of three studies conducted in Japan (12,223 patients from the CREDO-Kyoto registry cohort-2, RESET and NEXT), the DAPT score successfully stratified ischemic and bleeding risks, although the ischemic event rate was remarkably low even with high-DAPT score [214].

In 2019, ARC-HBR (Academic Research Consortium for High Bleeding Risk) criteria were proposed to identify the patients at HBR undergoing PCI [215]. Natsuaki et al. applied the ARC-HBR criteria in the CREDO-Kyoto Cohort-2 registry and reported the favorable performance of identifying the HBR patients [216]. Following this, Japanese Circulation Society proposed the Japanese-version HBR (J-HBR) criteria, adding heart failure, peripheral artery disease, low body weight, and frailty to the original ARC-HBR criteria [217]. Natsuaki et al. evaluated the performance of J-HBR criteria in the CREDO-Kyoto Cohort-3 registry, and reported J-HBR is useful in identifying the HBR patients undergoing PCI [218].

#### DAPT duration after DES implantation

Recent trials demonstrated the safety and efficacy of short DAPT followed by P2Y12 inhibitor monotherapy in ACS patients.

In the GLOBAL LEADERS trial, 1-month DAPT followed by ticagrelor monotherapy (experimental group) and 12-month DAPT (reference group) were compared [219]. In 7487 patients with ACS, the primary outcome of death or new Q wave MI occurred in 55 patients (1.5%) in the experimental group and in 75 patients (2.0%) in the reference group between 31 and 365 days after randomization (HR 0.73; 95% CI 0.51–1.03; *P* = 0.07) [220]. BARC 3 or 5 bleeding happened in 28 patients (0.8%) in the experimental group and in 54 patients (1.5%) in the reference arm (HR 0.52; 95% CI 0.33–0.81; *P* = 0.004).

Recently, ULTIMATE-DAPT trial compared 1-month DAPT (aspirin + ticagrelor) followed by ticagrelor monotherapy and 12-month DAPT (aspirin + ticagrelor) [150]. 3,400 patients with ACS (STEMI, NSTEMI, or UA) who were included in IVUS-ACS trial and had no events at 1-month follow-up were included in this trial. At 12 months, clinically relevant bleeding events (BARC type 2, 3 or 5) occurred less frequently in 1-month DAPT than in 12-month DAPT group (2.1% vs 4.6%; hazard ratio 0.45; 95% CI 0.30–0.66; *P* < 0.0001). The incidence of MACCE (a composite of cardiac death, MI, ischemic stroke, definite stent thrombosis, or clinically driven target-vessel revascularization) did not differ significantly between the groups (3.6% vs 3.7%; absolute risk difference −0.1%; 95% CI −1.4% to 1.2%; *P*_non-inferiority_ < 0.0001, *P*_superiority_ = 0.89).

These findings suggested that between 1 and 12 months after PCI in ACS, aspirin was associated with increased bleeding risk and appeared not to add to the benefit of ticagrelor on ischemic events.

In the SMART-CHOICE trial [221], 1498 patients were randomized to either DAPT for 3 months followed by P2Y12 inhibitor (clopidogrel, prasugrel, or ticagrelor) monotherapy or DAPT for 12 months, in which 314 STEMI and 469 NSTEMI patients were included. The rate of BARC 2–5 bleeding was significantly lower in the P2Y12 inhibitor monotherapy group than in the DAPT group (2.0% vs 3.4%, HR 0.58, 95% CI 0.36–0.92, *P* = 0.02), and MACE rates were similar (2.9% vs 2.5%). The TWILIGHT trial examined the effect of ticagrelor alone after 3-month DAPT vs ticagrelor plus aspirin among patients at high risk for bleeding or ischemic events after PCI [222]. Among patients with NSTE-ACS (*n* = 4614), ticagrelor monotherapy reduced BARC 2, 3, or 5 bleeding by 53% (3.6% vs 7.6%, HR 0.47, 95% CI 0.36–0.61, *P* < 0.001). Rates of all-cause death, MI, or stroke were similar (4.3% vs 4.4%, HR 0.97, 95% CI 0.74–1.28, *P* = 0.84) [223]. The TICO trial also compared ticagrelor monotherapy after 3-month DAPT vs 12-month DAPT [224]. In 1103 STEMI patients, ticagrelor monotherapy significantly reduced TIMI major bleeding (HR 0.32, 95% CI 0.12–0.87) without a significant increase in MACE (HR 1.10, 95% CI 0.53–2.27). In 1027 NSTEMI patients, ticagrelor monotherapy tended to reduce TIMI major bleeding (HR 0.69, 95% CI 0.34-0.143) and MACE (HR 0.58, 95% CI 0.30–1.13) [225]. These results corroborate the potential benefit of ticagrelor monotherapy after short DAPT in ACS patients.

Regarding the comparison between potent P2Y12 inhibitors, the ISAR-REACT 5 trial compared prasugrel plus aspirin vs ticagrelor plus aspirin in ACS patients, and demonstrated that treatment with prasugrel, compared to ticagrelor, significantly reduced the composite rate of death, MI, or stroke (6.9% vs 9.3%, *P* = 0.006) without any increase in bleeding complications (4.8% vs 5.4%, *P* = 0.46) [56].

MASTER-DAPT trial compared with 1-month DAPT and at least 6-month DPAT (at least 3-month DAPT for patients receiving anticoagulation) in high-bleeding risk population, in which ACS patients were included. The rates of both net adverse clinical events (NACE) and major adverse cardiac or cerebrovascular events (MACCE) were similar (7.5% vs 7.7% and 6.1% vs 5.9%) and met the trial definition for non-inferiority. The rate of major and clinically relevant nonmajor bleeding was significantly lower in the abbreviated 1-month DAPT group, compared to the prolonged DAPT group (6.5% vs 9.4%, *P* < 0.001). [226, 227]

It is well known that aspirin induces gastrointestinal ulceration and erosion [228]. In the Management of Aspirin-induced Gastrointestinal Complications (MAGIC) study, patients receiving PPI had lower risk of gastrointestinal ulcer or erosion [229, 230] Therefore, PPI should be more constantly used in patients with aspirin to reduce gastrointestinal toxicity during long-term prevention of cardiovascular events.

#### DAPT duration after DCB

As mentioned above (section of DCB), REC-CAGEFREE II trial reported the non-inferiority of 1-month DAPT after paclitaxel-coated balloon treatment in ACS patients compared with 12-month DAPT regarding net adverse clinical event.

#### DAPT dosage in Japan

Both prasugrel and ticagrelor are available, but the dose of prasugrel is different in Japan. While 60 mg loading dose and 10 mg maintenance dose are applied in Europe and US, 20 mg loading dose and 3.75 mg maintenance dose are used in Japan. Although clopidogrel is dominantly used around the world, smaller dose of prasugrel including loading confers a lower rate of bleeding events without an associated increase in ischemic events in Japan [53] (Table 1).

### Evidence from Japan

#### Antiplatelet therapy within 1 month after PCI

As described above, the ASET-JAPAN pilot study showed feasibility of prasugrel monotherapy with Japanese adjusted dose (3.75 mg/day) after SYNERGY stent implantation in Japanese CCS patients with anatomical SYNTAX Score < 23 [58]. The favorable 1-month outcomes in NSTE-ACS cohort were presented at EuroPCR 2024, and the 1-year results will be presented soon. The STOPDAPT-3 trial failed to show the superiority of prasugrel monotherapy for coprimary bleeding endpoint at 1 month compared with 1-month DAPT in patients with ACS or HBR [59]. We should acknowledge some important differences between ASET-Japan and STOPDAPT-3 studies. First, aspirin was loaded before PCI in ASET-Japan study whereas aspirin was not loaded in the monotherapy group in STOPDAPT-3. Second, the inclusion criteria for the two trials differed, with STOPDAPT-3 trial including more complex patients. We need to further investigate the potential population who would benefit from monotherapy without aspirin. Furthermore, the optimal dose of P2Y12 inhibitor when it is used as a monotherapy without aspirin after PCI needs to be elucidated.

The ongoing PREMIUM trial (NCT05709626) is investigating the safety of prasugrel monotherapy compared with 12-month DAPT in STEMI patients.

### Antiplatelet therapy after 1 month

The STOPDAPT 2 trial randomized 3,045 patients either to 1 month of DAPT followed by clopidogrel monotherapy or 12 months of DAPT with aspirin and clopidogrel [231]. One-month DAPT was superior to 12-month DAPT for the composite primary endpoint (cardiovascular death, MI, definite stent thrombosis, ischemic or hemorrhagic stroke, or TIMI major or minor bleeding) at 1 year, occurring in 2.36% with 1-month DAPT and 3.70% with 12-month DAPT (HR 0.64, 95% CI 0.42–0.98, *P* = 0.04). TIMI major or minor bleeding occurred in 0.41% with 1-month DAPT and 1.54% with 12-month DAPT (HR 0.26, 95% CI 0.11–0.64, *P* = 0.004). In the STOPDAPT 2 ACS trial, 4169 ACS patients were randomized and analyzed with the same antiplatelet regimen [232]. The same primary endpoint as the STOPDAPT 2 trial were applied to ACS patients, and cumulative event rates were 3.2% in the 1-month DAPT group and 2.83% in the 12-month group, which did not meet the statistical significance for non-inferiority (HR 1.14, 95% CI 0.80–1.62, *P*_non-inferiority_ 0.06), although cumulative rates of TIMI major and minor bleeding were significantly lower with 1-month DAPT (0.54% vs 1.17%, HR 0.46, 95% CI 0.23–0.94). After the publication of the STOPDAPT-2 and STOPDAPT-2 ACS trials, the results of prespecified pooled population analysis of these 2 trials, STOPDAPT-2 Total Cohort, were reported [233]. In 4136 ACS patients, 1-month DAPT was associated with a numerically higher cardiovascular event rates compared with 12-month DAPT at 1 year follow-up, but it was not statistically significant (2.76% vs 1.86%; hazard ratio 1.50 [95% CI 0.99–2.27]; *P* = 0.053).

#### Antiplatelet therapy beyond 1 year

STOPDAPT-2 5-year follow-up study reported that 1-month DAPT was superior for a composite of cardiovascular outcomes (cardiovascular death, MI, stroke, or definite stent thrombosis) but not comparable for major bleeding (TIMI major or minor bleeding) compared with 12-month DAPT [234]. Landmark analysis at 1 year showed a numerically lower incidence of cardiovascular events in 1-month DAPT group, but the difference was not statistically significant (*P* = 0.06).

The PANTHER group conducted a patient-level meta-analysis of 7 trials comparing P2Y12 inhibitor versus aspirin monotherapy in patients with established CAD [235]. The risk of the primary outcome (a composite of cardiovascular death, MI, or stroke) was lower with P2Y12 inhibitor monotherapy compared with aspirin at 2 years (hazard ratio 0.88; 95% CI 0.79–0.97; *P* = 0.012). The risk of major bleeding was not significantly different (hazard ratio 0.87; 95% CI 0.70–1.09; *P* = 0.23) and net adverse clinical events were lower with P2Y12 monotherapy (hazard ratio 0.89; 95% CI 0.81–0.98; *P* = 0.02).

#### Patients with atrial fibrillation

ESC 2023 ACS guidelines recommend that in ACS patients with atrial fibrillation (AF), after a short period of triple therapy up to 1 week from the acute event, a combination therapy with direct oral anticoagulant (DOAC) and P2Y12 inhibitor (preferably clopidogrel) should be continued for 1 year, followed by DOAC monotherapy. However, for high thrombotic risk patients, a period of triple therapy can be extended up to 1 month (Class IIa).

A meta-analysis of four DOAC-based RCTs comparing double vs triple antithrombotic therapy (PIONEER AF-PCI, RE-DUAL PCI, AUGUSTUS, and ENTRUST AF-PCI) reported that the incidence of the bleeding endpoint was significantly lower in double compared with triple antithrombotic therapy [236–240]. However, there were increased risks of stent thrombosis and a trend for increased risk of MI with double antithrombotic therapy. In these 4 trials, approximately half of patients presented with ACS and more than 90% of patients received clopidogrel as a P2Y12 inhibitor. We should acknowledge that in these trials, a vitamin K antagonist was used in triple antiplatelet therapy.

MASTER-DAPT study comparing abbreviated and prolonged DAPT following Ultimaster stent™ implantation in high-bleeding risk (HBR) patients indicated that abbreviated therapy resulted in a lower incidence of major or clinically relevant nonmajor bleeding [226]. Furthermore, a substudy of MASTER-DAPT using clopidogrel in patients with oral anticoagulant (OAC) revealed that it is safe and beneficial to stop DAPT at 1 month in HBR patients with or without an indication for OAC, while an abbreviated antiplatelet therapy strategy significantly reduced clinically relevant bleeding risk in HBR patients without OAC, but no such significant reduction was obtained in the OAC population [227].

The AFIRE trial demonstrated that DOAC monotherapy was non-inferior to combination therapy with DOAC and single antiplatelet therapy for efficacy (stroke, systemic embolism, MI, unstable angina requiring revascularization, or all-cause death; HR 0.72, 95% CI 0.55–0.95) and superior for safety (major bleeding; HR 0.59, 95% CI 0.39–0.89) in patients with atrial fibrillation and stable coronary artery disease including prior PCI more than 1 year earlier [241].

Although four major DOAC studies (i.e., PIONEER AF-PCI, RE-DUAL PCI, AUGUSTUS, and ENTRUST AF-PCI) clearly indicated the superiority of DOAC over warfarin, patients with impaired kidney function were excluded from such trials, because DOAC are not recommended in patients with significant renal dysfunction. To address such real-world limitations, Ozaki and his colleagues performed the REWRAPS study (NCT02024230) involving all comers regardless of kidney function [242]. While all the patients had coronary stenting and AF in the REWRAPS study, 250 patients were assigned to rivaroxaban and 245 patients were allocated to warfarin associated with a minimum 3-year follow-up. The trial is ongoing, and the results will be announced in the near future.

### Recommendations

- Short DAPT (1 month) followed by a potent P2Y12 inhibitor (possibly prasugrel or ticagrelor) monotherapy should be considered after PCI in patients with high-bleeding risk irrespective of complexity of coronary artery disease.
- One-month DAPT followed by clopidogrel monotherapy may not be recommended in patients with ACS.
- In patients without high-bleeding risks who are free from events after 3–6 months of DAPT, single antiplatelet therapy (preferably prasugrel or ticagrelor) should be considered.
- To reduce the risk of bleeding, a de-escalation strategy from prasugrel/ticagrelor to clopidogrel can be considered after 30 days from ACS.
- Prolonged DAPT (at least 6 months) should only be considered for patients with high thrombotic risk such as stent thrombosis without high-bleeding risk.
- In patients with atrial fibrillation and high-bleeding risk, triple antithrombotic therapy with DOAC, aspirin, and clopidogrel should be given in a short period up to 1 week followed by double therapy using DOAC and clopidogrel for 6 months, while in those with atrial fibrillation and high ischemic risk, triple antithrombotic therapy including DOAC, aspirin, and clopidogrel should be given up to 1 month followed by double therapy consisting of DOAC and clopidogrel for 12 months then DOAC monotherapy after the 12 months.
- A proton pump inhibitor (PPI) in combination with DAPT is recommended in patients at high risk of gastrointestinal bleeding.
- In patients with LV thrombus, anticoagulation should be administered for at least 6 months guided by repeated ultrasound or CT/MRI imaging.

## Multivessel disease and treatment of non-infarct-related artery

### Hemodynamically stable STEMI patients with multivessel disease

#### Complete revascularization vs culprit-only revascularization

#### Angiography-guided complete revascularization vs culprit-only revascularization

In the ESC 2023 guideline, for hemodynamically stable STEMI patients with multivessel disease, complete revascularization is recommended either during the index PCI or within 45 days as Class I with evidence level A. In the COMPLETE trial, 4041 patients with ST-segment elevation MI and multivessel disease who underwent primary PCI were randomized in a 1:1 fashion either to complete revascularization of non-infarct-related coronary arteries or no further revascularization [243]. In this trial, most of the non-culprit lesions were evaluated angiographically and less than 1% lesions were evaluated with FFR. In the complete revascularization group, PCI of non-culprit lesions was performed either during the index hospitalization or after discharge no later than 45 days from randomization. At a median follow-up period of 3 years, FFR-guided complete revascularization significantly reduced cardiovascular death or MI (7.8% vs 10.5%, HR 0.74, 95% CI 0.60–0.91, *P* = 0.004). In addition, there was no differential effect of the timing of PCI.

The benefit of complete revascularization for reducing cardiovascular death was also confirmed by a meta-analysis (odds ratio 0.69, 95% CI 0.48–0.99) [244].

#### FFR-guided complete revascularization vs culprit-only revascularization

DANAMI-3-PRIMULTI trial randomized 627 STEMI patients with multivessel disease after successful PCI of infarct-related artery either to FFR-guided complete revascularization before discharge or no further invasive treatment [245]. The primary endpoint was a composite of all-cause death, MI, or ischemia-driven revascularization of non-infarct-related arteries. During a median follow-up period of 27 months, FFR-guided complete revascularization significantly reduced the risk of the primary endpoint (13% vs 22%; hazard ratio 0.56; 95% CI 0.38–0.83; *P* = 0.004). The difference was mainly driven by ischemia-driven revascularization.

In the Compare-Acute trial, 885 patients with ST-segment elevation MI and multivessel disease who underwent primary PCI were randomized in a 1:2 fashion to complete revascularization of non-infarct-related coronary arteries guided by FFR or no revascularization of non-infarct-related coronary arteries [246, 247]. There was a significant reduction in MACE at 3 years with FFR-guided complete revascularization (15.6% vs 30.2%, HR 0.46, 95% CI 0.33–0.64, *P* < 0.001). The benefit was mostly driven by a reduced risk of revascularization.

Recently, two trials have compared the FFR-guided complete revascularization and culprit-only revascularization in patients with AMI and multivessel disease.

FIRE randomized trial investigated the benefit of FFR-guided complete revascularization in 1445 older patients (≧75 years old) presenting with AMI and multivessel disease compared with culprit-only strategy [248]. Thirty-five percent of patients presented with STEMI and 65% with NSTEMI. FFR-guided complete revascularization significantly reduced the rate of the primary outcome (a composite of death, MI, stroke, or ischemia-driven coronary revascularization) at 1 year (15.7% vs 21.0%; hazard ratio 0.73; 95% CI 0.57–0.93; *P* = 0.01). The occurrence of death or MI was also lower in the FFR-guided group than in the culprit-only group (18.3% vs 12.9%; hazard ratio 0.68; 95% CI 0.52–0.88).

FULL REVASC trial compared the FFR-guided complete revascularization and culprit-only strategy in 1542 patients with STEMI or very-high-risk NSTEMI and multivessel disease [249]. More than 90% were patients with STEMI and the mean age was 65 years. FFR-guided complete revascularization did not reduce the risk of the primary endpoint (a composite of all-cause death, MI, or unplanned revascularization) at a median follow-up period of 4.8 years (19.0% vs 20.4%; hazard ratio 0.93; 95% CI 0.74–1.17; *P* = 0.53). The incidence of any restenosis, stent thrombosis, and target-vessel revascularization were higher in the FFR-guided group than in the culprit-only group. It should be noted that the recruitment of patients in this trial was terminated prematurely after the publication of the COMPLETE trial [243].

#### FFR-guided vs angiography-guided complete revascularization in hemodynamically stable STEMI patients with multivessel disease

In the ESC 2023 guidelines for the management of ACS, the decision of PCI of the non-infarct-related artery in STEMI patients is recommended to be based on angiographic assessment (Class I, evidence level B).

As mentioned above, the Compare-Acute and COMPLETE trials applied fractional flow reserve (FFR) guide assessment of non-infarct-related artery for complete revascularization in STEMI patients, and in both trials, FFR-guided complete revascularization significantly reduced cardiovascular events, compared to culprit-lesion-only PCI [243, 246].

Direct comparison between FFR-guided and angiography-guided complete revascularization was performed in the FUTURE trial; however, this trial was terminated prematurely, because the safety analysis showed significantly higher occurrence of death in the FFR-guided group [250]. Approximately half of the patients presented with ACS and the FFR-guided strategy did not reduce a composite of major vascular adverse cardiovascular events at 1 year.

FLOWER-AMI compared angiography-guided and FFR-guided complete revascularization in STEMI patients with multivessel disease [251]. The primary outcome was a composite of death from any cause, nonfatal myocardial infarction, or unplanned hospitalization leading to urgent revascularization at 1 year, and a primary outcome event occurred in 32 of 586 patients (5.5%) in the FFR-guided group and in 24 of 577 patients (4.2%) in the angiography-guided group (HR, 1.32; 95% CI, 0.78–2.23; *P* = 0.31). Although an FFR-guided strategy failed to show a significant benefit over an angiography-guided strategy, considering the wide confidence intervals for the estimate of effect, we still need the evidence for physiological assessment of non-infarcted-related artery in acute setting.

The FRAME-AMI trial compared FFR-guided vs angiography-guided complete revascularization in AMI patients with multivessel disease [252]. 562 AMI patients who underwent successful PCI of the infarct-related artery were randomized to either FFR-guided or angiography-guided PCI for non-infarct-related artery. FFR-guidance significantly reduced the occurrence of the primary endpoint (a composite of time to death, MI, or repeat revascularization) at a median follow-up period of 3.5 years (7.4% vs 19.7%; hazard ratio, 0.43; 95% CI 0.25–0.75; *P* = 0.003). The benefit of an FFR-guided strategy was mainly observed in patients presenting with NSTEMI. It should be noted that this trial recruited less than half the planned number of patients due to slow patient recruitment.

Barauskas et al*.* reported the single-center randomized study comparing QFR-guided and angiography-guided PCI of non-infarct-related artery in 198 patients with STEMI [253]. QFR-guided strategy reduced the mortality and revascularization at 1 year; however, this study lacks the detailed methodology (e.g., sample size calculation, randomization scheme, etc.).

#### Timing of revascularization of non-infarct-related artery

As described above, in the COMPLETE trial, the complete revascularization group performed PCI of non-culprit lesion either during the index hospitalization or after discharge no later than 45 days from randomization [243]. Considering this, the ESC 2023 guideline recommends complete revascularization either during the index PCI or within 45 days for hemodynamically stable STEMI patients with multivessel disease (Class I, evidence level A).

Recently, the BIOVASC trial was published in 2023 [254]. This trial randomized 1525 patients with ACS (STEMI or NSTE-ACS) and multivessel disease either to immediate or staged complete revascularization group. The immediate complete revascularization was non-inferior to staged complete revascularization in terms of 1-year primary endpoint (composite of all-death, MI, any unplanned ischemia-driven revascularization or cerebrovascular events; 7.6% vs 9.4%; HR 0.78; 95% CI 0.55–1.11, *P*_non-inferiority_ = 0.0011). There was no differential impact between STEMI and NSTE-ACS patients for the primary outcome. The incidence of the composite endpoint at 30 days, and MI and unplanned revascularization at 1 year were significantly lower in immediate than in staged group.

More recently, MULTISTARS AMI trial, which randomized 840 STEMI patients with multivessel disease either to undergo immediate PCI for the non-culprit lesion or to undergo staged PCI within 19–45 days after the index procedure, reported the superior result of immediate group for the primary composite endpoint (all-death, nonfatal MI, stroke, unplanned ischemia-driven revascularization, or heart failure hospitalization) at 1 year (8.5% vs 16.3%; risk ratio 0.52; 95% CI 0.38–0.72; *P* < 0.001 for superiority) [255]. The difference was mainly driven by MI (2.0% vs 5.3%) and unplanned ischemia-driven revascularization (4.1% vs 9.3%).

These 2 recently published data, which are not included in the ESC 2023 guidelines, support the immediate PCI in patients with stable STEMI with multivessel disease. It should be noted that in BIOVASC trial, FFR or iFR were used for the assessment of non-culprit lesion in approximately 20% of patients, and in MULTISTARS AMI trial, the assessment was performed only angiographically.

### Hemodynamically stable NSTE-ACS patients with multivessel disease

#### Complete vs culprit-only revascularization

The evidence for patients with NSTE-ACS and multivessel disease is lacking. In a retrospective observational study in UK, 21,857 patients with NSTEMI and multivessel disease, including 11,737 (54%) patients who underwent single-stage complete revascularization and 10,120 patients who underwent culprit-only revascularization, were investigated [256]. Although having more high-risk backgrounds, single-stage complete revascularization group had a lower mortality during a median follow-up period of 4 years (22.5% vs 25.9%, *P* = 0.0005). The ESC 2023 guideline recommends complete revascularization, preferably during the index procedure in patients presenting with hemodynamically stable NSTE-ACS with multivessel disease, as Class IIa with evidence level C. Dedicated randomized trials are needed to investigate the benefit of complete revascularization over culprit-only revascularization in NSTE-ACS patients with multivessel disease.

The FAMOUS-NSTEMI trial randomized NSTEMI patients with at least one coronary stenosis (> 30%) to angiography-guided or FFR-guided strategy [257]. The primary outcome was the proportion of patients allocated to medical treatment. More than half of the patients had multivessel disease defined as angiographic stenosis more than 50%. The proportion of patients treated by medical therapy was higher in the FFR-guided group than in the angiography-guided group (22.7% vs 13.2%; 95% CI 1.4–17.7%; *P* = 0.022). The occurrence of revascularization was lower in the FFR-guided group at 1 year. As described above, FRAME-AMI trial reported the favorable outcome of FFR-guided strategy than angiography-guided strategy in patients with AMI and multivessel disease, which was mainly driven by NSTEMI population [252].

#### Timing of revascularization of non-culprit lesion

Recently, the substudy of BIOVASC trial in patients with NSTE-ACS was reported [258]. In 917 NSTE-ACS patients with multivessel disease who were randomized either to immediate or staged complete revascularization, the incidence of the primary endpoint (mentioned above) at 1 year was similar between the two groups. However, immediate complete revascularization was associated with reduced incidence of MI (2.0% vs 5.3%; risk difference 3.3%; 95% CI 0.9–5.7; *P* = 0.006), and unplanned revascularization (4.2% vs 7.8%; risk difference 3.5%; 95% CI 0.4–6.6; *P* = 0.018) at 1 year.

### ACS patients with multivessel disease presenting in cardiogenic shock

In the setting of cardiogenic shock, the efficacy and safety of treating non-infarct-related coronary arteries in the context of primary PCI has been a matter of debate. In the CULPRIT-SHOCK (Culprit-Lesion-Only PCI versus Multivessel PCI in Cardiogenic Shock) trial (*N* = 706), the 30-day risk of a composite of death or severe renal failure leading to renal-replacement therapy was lower in patients who underwent initial PCI of the culprit lesion only compared with those who underwent immediate multivessel PCI [259]. Between 30 days and 1 year, there was no significant difference in all-cause death between the two groups [260]. Based on this evidence, for patients with ACS complicated by cardiogenic shock, immediate PCI of infarct-related artery only during the index procedure is recommended as Class I with evidence level B. However, a patient-pooled analysis from RESCUE and SMC-ECMO registries reported that in 315 AMI patients with advanced shock requiring VA-ECMO before PCI, the immediate multivessel PCI reduced the incidence of 30-day mortality or renal-replacement therapy (68.0% versus 54.3%; *P* = 0.018) and 12-month mortality (59.5% versus 47.5%; hazard ratio 0.69 [95% CI, 0.51–0.94]; *P* = 0.018) compared with culprit-only PCI [261]. Dedicated trials regarding the timing of PCI for the patients with advanced shock with ACS and multivessel disease requiring mechanical support are needed.

### Recommendations

- Complete revascularization should be considered in STEMI or NSTEMI patients with multivessel disease.
- In patients with stable STEMI with multivessel disease, complete revascularization either during the index PCI or within 45 days is recommended.
- In patients with stable NSTE-ACS with multivessel disease, complete revascularization preferably during the index procedure is recommended.
- Non-infarct-related artery PCI during the index procedure is not recommended in patients with cardiogenic shock.

## Myocardial infarction with non-obstructive coronary arteries (MINOCA)

MINOCA is an emerging topic in the field of cardiology. The consensus paper/guidelines of ESC [262], AHA [263], or JCC/CVIT [264] are available and well summarized by Takahashi et al [265]. The definition of MINOCA is (1) fulfilling the criteria of AMI with elevated cardiac biomarker (preferably cardiac troponin), (2) absence of obstructive CAD on angiography (< 50% stenosis), and (3) absence of any obvious other cause. The prevalence of MINOCA differs across the studies, ranging 1–14% of patients with suspected ACS [266]. The potential causes of MINOCA include plaque rupture/erosion, coronary artery spasm, spontaneous coronary dissection, coronary microvascular disorders, etc. As MINOCA falls under the category of “myocardial infarction”, its cause must be myocardial ischemia, and it should be distinguished from non-ischemic myocardial injury (e.g., myocarditis, Takotsubo cardiomyopathy, pulmonary embolism, sepsis, etc.). In clinical practice, it is not practical to immediately diagnose MINOCA when significant stenosis is not observed on angiography, since there are many potential causes of an elevated troponin. Therefore, in the acute phase of patients with suspected AMI without significant stenosis, MINOCA should be regarded as a working diagnosis that requires further investigation rather than a final diagnosis based solely on this point. For more details on MINOCA including a diagnostic algorithm, please refer to other sources such as guidelines, consensus documents or research papers.

## Summary

**Fig. 3:**
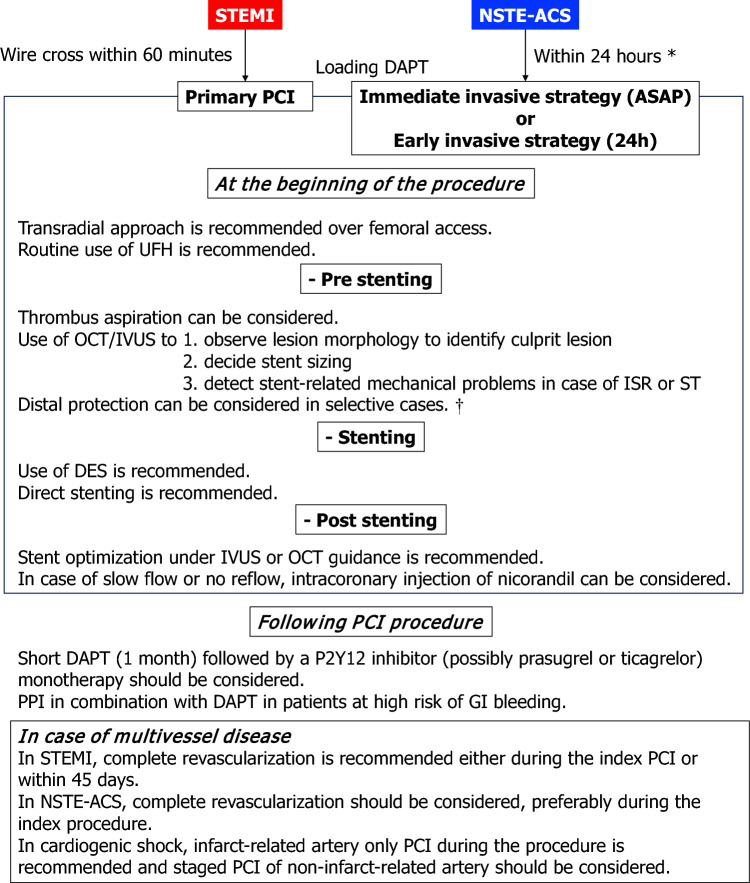
Summary of recommendations in PCI for ACS. *An immediate invasive (as soon as possible) strategy is recommended in NSTE-ACS patients with very high-risk criteria. An early invasive strategy (within 24 h) should be considered in NSTE-ACS patients with high-risk criteria. †Cases with large thrombus formation or plaque burden with a high possibility of distal embolism or slow/no flow; or cases with MI in SVG. *DAPT* dual antiplatelet therapy; *DES* drug-eluting stent; *GI* gastrointestinal; *ISR* in-stent restenosis; *IVUS* intravascular ultrasound; *NSTE-ACS* non-ST-segment elevation acute coronary syndrome; *OCT* optical coherence tomography; *PCI* percutaneous coronary intervention; *PPI* proton pump inhibitor; *ST* stent thrombosis; *STEMI* ST-segment elevation myocardial infarction; *UFH * unfractionated heparin

The Task Force on Primary PCI of the CVIT society has updated this expert consensus document for the management of ACS in 2024 version based on new evidence (Fig. 3). Our team would like to recommend the following strategies in ACS: (1) in patients with STEMI, primary PCI should be initiated within 120 min, preferably wire crossing done within 60 min; (2) in NSTE-ACS patients at very high risk, an immediate invasive strategy (as soon as possible) is recommended; (3) in NSTE-ACS patients at high risk, early invasive strategy (within 24 h) is recommended; (4) radial access and drug-eluting stent (DES) over bare-metal stent (BMS) are recommended; (5) complete revascularization (either immediate or staged) is preferred in hemodynamically stable ACS patients with multivessel disease.

Intravascular imaging is recommended to guide optimal PCI. Intravascular imaging is also useful to identify the culprit lesion when it is not evident angiographically in NSTE-ACS. Thrombus detection, where OCT/OFDI is the current gold standard, facilitates identification of an ACS culprit lesion.

Earlier studies have shown the benefit of thrombus aspiration in primary PCI; however, routine use of mechanical thrombus aspiration is no longer recommended in Europe due to the safety concerns regarding the risk of stroke. However, there are several studies in Japan showing the benefit of thrombus aspiration in primary PCI. Therefore, in the absence of GP IIb/IIIa inhibitors, thrombus aspiration may be considered in primary PCI.

Concerning the duration of antiplatelet therapy, short DAPT (1 month) followed by P2Y12 inhibitor has become the first choice in patients with high-bleeding risk. Furthermore, although clopidogrel is dominantly used around the world, a smaller maintenance dose of prasugrel including a loading dose confers fewer bleeding complications associated without increased risk of ischemic events in Japan. In patients with atrial fibrillation and high-bleeding risk, following a short period of triple antithrombotic therapy (up to 1 week from the acute event), dual antithrombotic therapy (e.g., DOAC and single oral antiplatelet agent preferably clopidogrel) is recommended with cessation of antiplatelet therapy after 6 months. In patients with atrial fibrillation and high ischemic risk, triple antithrombotic therapy, including DOAC, aspirin, and clopidogrel, should be given up to 1 month followed by double therapy consisting of DOAC and clopidogrel for 12 months and then DOAC monotherapy after the 12 months.

While the Compare-Acute and COMPLETE trials applied FFR-guided assessment of non-infarcted-related artery for complete revascularization in STEMI patients, FFR-guided complete revascularization significantly reduced cardiovascular events compared to culprit-lesion-only PCI. However, the FLOWER-MI study performed a direct comparison between angiography-guided and FFR-guided complete revascularization and failed to show a significant benefit over an angiography-guided strategy. We still need evidence for physiological assessment of non-infarcted-related artery in the acute setting. Furthermore, in the near future, such physiological assessment may favor less invasive approaches such as QFR or FFR-CT especially in non-culprit vessels.

## Data Availability

The data that support the findings of this study are available from the corresponding author with the agreement of CVIT Steering Council upon reasonable request.
